# Acute phase reactions in *Daboia siamensis* venom and fraction-induced acute kidney injury: the role of oxidative stress and inflammatory pathways in *in vivo* rabbit and *ex vivo* rabbit kidney models

**DOI:** 10.1590/1678-9199-JVATITD-2023-0070

**Published:** 2024-05-20

**Authors:** Narongsak Chaiyabutr, Jureeporn Noiprom, Kanyanat Promruangreang, Taksa Vasaruchapong, Panithi Laoungbua, Orawan Khow, Lawan Chanhome, Visith Sitprija

**Affiliations:** 1 Queen Saovabha Memorial Institute, The Thai Red Cross Society, Pathumwan, Bangkok, Thailand.; 2 Department of Research and Development, Queen Saovabha Memorial Institute, The Thai Red Cross Society, Bangkok, Thailand.; 3 Snake Farm, Queen Saovabha Memorial Institute, The Thai Red Cross Society, Bangkok, Thailand.

**Keywords:** *Daboia siamensis* venom, Venom fractions, Acute kidney injury, Oxidative stress, Inflammatory cytokines, Rabbits

## Abstract

**Background:**

This study examines the direct nephrotoxic effects of *Daboia siamensis* venom (RVV) and venom fractions in *in vivo* and isolated perfused kidneys (IPK) to understand the role of inflammation pathways and susceptibility to oxidative stress in venom or fraction-induced acute renal failure.

**Methods:**

We administered RVV and its venom fractions (PLA_2_, MP, LAAO, and PDE) to rabbits *in vivo* and in the IPK model. We measured oxidative stress biomarkers (SOD, CAT, GSH, and MDA) in kidney tissue, as well as inflammatory cytokines (TNF-α, IL-1β, IFN-γ, IL-4, IL-5, and IL-10), MDA and GSH levels in plasma and urine. We also calculated fractional excretion (FE) for pro-/anti-inflammatory cytokines and oxidative stress biomarkers, including the ratios of pro-/anti-inflammatory cytokines in urine after envenomation.

**Results:**

In both kidney models, significant increases in MDA, SOD, CAT, and GSH levels were observed in kidney tissues, along with elevated concentrations of MDA and GSH in plasma and urine after injecting RVV and venom fractions. Moreover, RVV injections led to progressive increases in FE_MDA_ and decreases in FE_GSH._ The concentrations of IL-4, IL-5, IL-10, IFN-γ, and TNF-α in plasma increased *in vivo*, as well as in the urine of the IPK model, but not for IL-1β in both plasma and urine after RVV administrations. Urinary fractional excretion of TNF-α, IL-1β, IFN-γ, IL-4, IL-5, and IL-10 tended to decrease *in vivo* but showed elevated levels in the IPK model. A single RVV injection *in vivo* disrupted the balance of urinary cytokines, significantly reducing either the TNF-α/IL-10 ratio or the IFN-γ/IL-10 ratio.

**Conclusion:**

RVV induces renal tubular toxicity by increasing oxidative stress production and elevating inflammatory cytokines in urine. During the acute phase of acute kidney injury, the balance of urine cytokines shifts toward anti-inflammatory dominance within the first two hours post-RVV and venom fractions.

## Background

Snakebite envenoming is a significant public health issue and it is recognized as a neglected tropical disease by the World Health Organization (WHO) [1]. Among the venomous snakes, *Daboia siamensis* (*D. siamensis*), a member of the Russell's viper subspecies, holds great importance. It is found in various Southeast Asian countries, such as Myanmar, Thailand, and Vietnam, and is responsible for numerous clinical cases of envenomation, leading to a significant number of deaths, and systemic envenoming complications including acute kidney injury (AKI) [2]. However, the current understanding of the inflammatory responses and their association with AKI is still limited, particularly on the pathophysiological mechanisms of *D. siamensis* venom (RVV) induced acute renal failure.

RVV consists of a complex mixture of specific enzymatic and non-enzymatic toxins, which induce a wide range of pathophysiological events, leading to both local and systemic clinical changes. These include intravascular hemolysis [3], disseminated intravascular coagulation with or without microangiopathy coagulation disorders [4], hemodynamic changes resulting in hypotension and circulatory collapse [5], as well as direct nephrotoxicity of the venom [6, 7]. RVV-induced nephrotoxicity effects are characterized by the deterioration of renal function, manifested by disturbances in electrolyte and acid-base homeostasis, occurring within hours, days, or even weeks [8]. Other factors that may contribute to the development of renal injury after envenomation are associated with secondary complications, especially oxidative stress caused by venom toxins, which may persist even after antivenom administration [9], or can appear within months or years after envenomation [8]. The timing of antivenom administration and administration of inadequate antivenom have been shown to be possible factors contributing to the development of renal injury after envenomation. Delaying the administration of antivenom beyond 1-3 hours after snakebite increases the risk of AKI [10]. A study showed that antivenom administration within 3-6 hours after Russell's viper bite could not prevent AKI but could reduce the severity of renal damage [11]. Clinical and experimental animal studies have demonstrated that renal inflammation is modulated by inflammatory mediators [12, 13]. It has been reported that RVV-induced AKI leads to irreversible renal damage and the development of chronic kidney disease (CKD) [14, 15]. Thus, there is no consensus on the single mechanism causing acute renal failure after a viper bite, particularly concerning the direct cytotoxic action of RVV at the intra-renal tissue level. Currently, there is limited understanding of the characteristics of early inflammatory responses within the first three hours after envenomation with RVV and their relationship with oxidative stress and inflammatory pathways in AKI. Snake venom has been shown to contribute to the generation of reactive oxygen species (ROS), causing oxidative stress [8, 9]. This, in turn, triggers inflammatory pathways and exacerbates renal dysfunction. Oxidative stress and inflammation are suspected to be secondary effects of snakebite and may play a central role in the progression of renal failure [8, 16].

The kidney, with its high blood flow and ability to concentrate substances in the urine, serves as the organ responsible for toxin removal. Oxidative damage and inflammatory mediator infiltration persist post-antivenin treatment for envenomation [9], posing a challenge for medical practitioners in managing viper bites as renal abnormalities may manifest months later [17]. Renal inflammation can be influenced by various inflammatory mediators, including cytokines, which play a crucial role in both initiating and sustaining inflammation [12, 13]. In cases of AKI, numerous cytokines are released by leukocytes and renal tubular cells in the injured kidney [18]. The pathophysiology of AKI, specifically the direct cytotoxic effects of venom on the kidneys, is suspected, but there is still a lack of convincing evidence regarding the integrated mechanisms of oxidative stress and inflammatory signaling in regulating renal pathophysiological function in relation to the venom's toxic components.

The present study aimed to investigate acute phase reactions associated with acute kidney injury (AKI) induced by RVV and its venom fractions in *in vivo* rabbit and IPK rabbit kidney models, thereby enhancing our understanding of envenomation pathophysiology. This study focused on understanding oxidative stress and inflammatory pathways, locally and systemically, following injection of RVV and its venom fractions containing phospholipase A_2_ (PLA_2_), metalloprotease (MP), L-amino acid oxidase (LAAO), and phosphodiesterase (PDE). The investigation included assessing kidney oxidant and inflammatory mediator concentrations, along with urinary biomarkers, and evaluating urinary fractional excretion of oxidants and inflammatory cytokines, including pro-/anti-inflammatory ratios in groups of rabbits injected with RVV and venom fractions. Inflammatory and oxidative stress biomarkers were analyzed in urine, plasma, and kidney tissue post-envenomation. The study also examined a cytokine panel in urine and plasma, covering pro-inflammatory cytokines [interferon gamma (IFN-γ), interleukin-1 beta (IL-1β), and tumor necrosis factor alpha (TNF-α)] and anti-inflammatory cytokines [interleukin 4 (IL-4), interleukin 5 (IL-5), and interleukin 10 (IL-10)], as well as oxidative stress markers in kidney tissue, including superoxide dismutase (SOD), catalase (CAT), glutathione-S-transferase (GSH), and malondialdehyde (MDA) levels. Additionally, MDA and GSH concentrations were measured in plasma and urine.

## Methods

### Animals

Adult male white New Zealand rabbits weighing 2 to 3 kg were employed for experimental trials involving both *in vivo* and *ex vivo* studies of isolated perfused rabbit kidneys (IPK). The animals were obtained from the Animal House at Queen Saovabha Memorial Institute (QSMI). They were housed in stainless steel cages, provided with a standard diet and water, exposed to a 12-hour light/dark cycle, and maintained at a laboratory temperature of 26 ± 1 °C. The animals were kept under observation in the Animal House for two weeks before the start of experiments. The experiments were conducted in compliance with the Ethics Committee of Queen Saovabha Memorial Institute Animal Care and Use, under the approval number QSMI ACUC-03-2016, following the guidelines of the National Research Council of Thailand.

### Snakes and venom sample collections

*D. siamensis*, native to the eastern regions of Thailand, was held in captivity at the Snake Farm of the Queen Saovabha Memorial Institute (QSMI) in Thailand. Each snake was housed individually in plastic cages and had unrestricted access to water in the animal care room at the Snake Farm. Once a month, these snakes were fed small rodents in accordance with their weight (10-20% of their body weight). All the snakes were kept under standard conditions of ambient temperature (averaging 27 °C) and relative humidity (75%). The venom from *D. siamensis* snakes was extracted and collected in glass vials through the snake's bite on a parafilm membrane covering a glass vessel. The fresh venom was then pooled, immediately frozen at -20 °C, and lyophilized using the Freeze Dryer Model FDL-10N-50-TD (MRC Scientific Instruments). The lyophilized venom was stored at -20 °C until use. A pool of RVV was acquired from 14 adults, comprising six males and eight females.

### Isolation of enzymatic compositions of RVV

The venom compositions of RVV were isolated for phospholipase A_2_ (PLA_2_) and metalloproteinase (MP) as the dominant protein families, and L-amino acid oxidase (LAAO) and phosphodiesterase (PDE) as minor protein families, as previously described [19]. Briefly, crude RVV was divided for isolation through fractionation methods. One hundred milligrams of pool-crude RVV were used to obtain phospholipase A_2_ (PLA_2_), resulting in a protein yield of 7.6 mg. Another 100 mg of pool-crude RVV was isolated using fractionation methods for metalloprotease (MP), L-amino acid oxidase (LAAO), and phosphodiesterase (PDE), yielding 4.8 mg, 0.7 mg, and 0.32 mg of protein, respectively, for comparative purposes. The enzymatic activities of crude RVV venoms were measured as previously described [19].

To isolate PLA_2_ from crude RVV, ion-exchange chromatography was performed on a HiTrap CMFF column (GE Healthcare, Sweden). PLA_2_ activity was assessed according to the method of Holzer and Mackessy [20]. The isolation of MP, PDE, and LAAO was achieved through gel filtration on SuperdexTM 75 10/300GL and column ion-exchange chromatography. The proteolytic activity and inhibitor assay for MP were determined using the method described by Anson [21]. LAAO activity was determined according to the Worthington Enzyme Manual [22]. PDE activity was measured according to Lo et al. [23].

### Dosage and administration regimen of venom

The concentrations of crude RVV used were based on a previous study in experimental animals, either dogs or rabbits, in which the dosage of RVV in lyophilized form caused death in 50% of subjects (LD50) by intravenous injection, and was determined to be 0.5 mg/kg body weight [5, 24]. However, a single venom dose of 0.1 mg/kg was arbitrarily chosen in the present work for rabbits, based on preliminary experiments in which doses of 0.1 mg/kg and 0.5 mg/kg were tested. Rabbits injected with 0.1 mg/kg showed minimal systemic and renal function alterations, whereas those receiving 0.5 mg/kg generally died within a few minutes or hours after venom administration. This short survival time precluded adequate assessment of changes in renal functions. Therefore, the dose of 0.1 mg/kg provided the best combination of renal damage (assessed through oxidative stress and cytokine study) in relation to survival time for 2-3 hours. Thus, a single venom dose of 0.1 mg/kg was used for intravenous injection in the *in vivo* study.

### Preparation of *in vivo* envenomed rabbit model

In the *in vivo* study, experiments were conducted using adult male white New Zealand rabbits. The animals were fasted for 12 hours with free access to water ad libitum before the experiment. On the day of the experiment, the animals were anesthetized by intravenous injection with pentobarbital sodium (50 mg/kg) via the marginal ear vein and were subsequently given small maintenance doses (10-20 mg) as necessary to provide a level of light state of anesthesia throughout the procedure. A tracheotomy was performed to ensure a clear airway using an endotracheal tube. Polyethylene tubes (PE 90) were used for cannulation of the jugular vein to allow infusion of the solution and facilitate renal clearance studies. Additionally, a polyethylene tube (PE 90) was inserted into the carotid artery to measure blood pressure and heart rate (recorded using a Polygraph Model 79, Grass Instruments Co.), as well as for arterial blood sampling. For urine collection, a polyvinyl catheter (PV 120) was inserted into the left ureter using a retroperitoneal approach. Urine samples and heparinized plasma samples were collected within each interval throughout the experimental period after the administration of RVV or one of its venom fractions. The carotid arterial blood sample, withdrawn in the amount of 1-1.5 mL, was placed in a 2 mL heparinized polypropylene tube with a snap-on cap. At the end of the experiment, the animal was euthanized by intravenous injection of an overdose of pentobarbital sodium via the marginal ear vein.

The renal hemodynamics in the *in vivo* study were assessed in experimental animals following the previously described protocol [5]. In brief, we administered a priming dose solution (0.5 mL/kg body weight) containing 5% inulin (In) and 1.2% p-amino hippuric acid (PAH) in 0.15 M NaCl at pH 7.4 through the jugular vein catheter. This was followed by a continuous infusion of a sustaining solution containing 0.5% inulin and 0.12% PAH in 0.15 M NaCl, delivered at a rate of 1.0 mL/min using a peristaltic pump (EYELA Microtube pump MP-3 Tokyo Rikakikai Co. Ltd.) throughout the experimental period. After a 30-minute equilibration period, we conducted the control period for kidney clearance studies and general circulation measurements as pretreatment before administration with specified lyophilized RVV and each venom fraction in their respective groups (four rabbits per group). Renal functions and general circulation were recorded at 0 (control), 10, 30, 60, 90, and 120 minutes after envenomation. Urine and arterial blood samples were collected at time intervals for inulin clearance, PAH clearance, oxidative stress, and inflammatory cytokines level determination.

### Isolated perfused rabbit kidney preparation

In the *ex vivo* study, the preparation of the IPK was based on the method previously described [24]. In brief, adult male white New Zealand rabbits were fasted for 12 hours prior to the experiment with access to water *ad libitum*. The rabbit was anesthetized by intravenous injection with pentobarbital sodium (50 mg/kg) via the marginal ear vein After careful dissection, the left kidney was prepared for perfusion, and a polyvinyl catheter was inserted into the left ureter for urine collection. The kidney's renal artery was directly cannulated with a 19-gauge stainless steel needle, approximately 1.0 inch in length with a smooth tip, and flushed immediately with heparinized saline (100 units/mL). The kidney, with the renal vein and ureter intact, was isolated and promptly transferred to a temperature-controlled tissue bath organ chamber (Radnoti, chamber for organ isolation procedures, catalog No. 166070, Grass Technologies, Monrovia, CA, USA). After the preparation of IPK, the animal was euthanized by intravenous injection with an overdose of pentobarbital sodium via the marginal ear vein. The IPK perfusion apparatus employed the previously described perfusion method [24]. The working perfusate, recirculating perfusion, consisted of a modified Krebs-Henseleit solution (MKHS) oxygenated at 37 °C with a 19:1 (v/v) mixture of O_2_:CO_2_. The perfusion was carried out through the renal artery using a recirculating rotary pump (EYELA, Roller pump, RP-1000). The kidney was allowed to stabilize in the perfusion system for 30 minutes to maintain a constant perfusion flow rate (40-60 ml/min) as indicated by the maintenance of urine flow (UF) and perfusion pressure (PP) at 100 mmHg. The MKHS preparation in 100 mL comprised: 141 mM Na^+^, 5.4 mM K^+^, 1.9 mM Ca^2+^, 2.4 mM Mg^2+^, 126 mM Cl^-^, 25 mM HCO_3_^-^, 2.44 mM SO_4_^2-^, 1.5 mM PO_4_^3-^, and 13 mM amino acids composed of twenty physiological amino acids [25]. The total perfusate included 100 mg of D-glucose and 50 mg of inulin, alongside oncotic agents. These agents were modified in our laboratory by adding 3 g of bovine serum albumin (fraction V, from Sigma Chemical Co., St. Louis, MO, USA) and 2 g of dextran (from Leuconostoc spp. Mr. 100,000, Sigma Chemical Co., St. Louis, MO, USA). The pH of the perfusion solution was adjusted to 7.4, and a 1.2 μm filter was connected to the perfusate coil inlet of the tissue bath organ chamber. Changes in PP within the kidney were measured at the tip of the stainless-steel cannula using a single-tube mercury manometer. PP measurements were taken at 5-minute intervals after equilibration. The initial 30 minutes of perfusion were designated as the internal control period. The experimental period was divided into time intervals of 0, 5, 10, 30, 60, and 90 minutes of perfusion time. The experiments were conducted over 90 minutes following the administration of RVV or one of its venom fractions. Within each interval, 1 mL of perfusate and urine sample was collected for five minutes and placed in a 2 mL polypropylene tube with a screw cap. They were stored at -70 ºC for further determinations of oxidative stress and cytokine parameters, including inulin clearance.

### Experimental design

*The first experimental trial*

Twenty adult male white New Zealand rabbits were divided into five groups of four animals each (n = 4/group). Group I received intravenous injections of lyophilized crude venom (0.1 mg/kg, i.v.) in 1 mL of 0.15 M NaCl. Group II received intravenous injections of venom fractions of PLA_2_ (0.2 mg/kg). Group III received intravenous injections of venom fractions of MP (0.2 mg/kg). In Group IV, animals were injected intravenously with venom fractions of LAAO (0.15 mg/kg). Group V received intravenous injections of venom fractions of PDE (0.1 mg/kg).

Experimental periods lasted for 120 minutes with each interval period, both plasma and urine samples were frozen in liquid nitrogen at -70 ºC for the determination of renal clearance, expression analysis of cytokine concentrations, and oxidative stress parameters. At the end of the experiment, animals were euthanized with a high dose of pentobarbital sodium. Subsequently, the left kidney was removed, and portions of the kidney were immediately immersed in liquid nitrogen, frozen, and stored at -70 ºC for further analysis of the activities of SOD and CAT, concentrations of reduced GSH and MDA in the kidney.

*The second experimental trial*

The objective was to study the effects of crude RVV and venom fractions (PLA_2_, MP, LAAO, and PDE) on renal functions in an *ex vivo* in the IPK model. The dosages of crude RVV and venom fractions used in the present study were chosen arbitrarily and adjusted based on a previous investigation in experimental animals and IPK, as described previously [19]. The experimental trials in IPK were divided into five experimental groups (n = 4 each/group) as follows: Group 1: The IPK was treated with 1 mL of lyophilized RVV in normal saline (1 mg/mL), which was added to 100 mL of perfusate in the recirculating system after a 30-minute equilibration period, serving as an internal control. Group 2: The IPK was treated only with 1 mL of venom fractions of PLA_2_ (280 μg/mL), added to 100 mL of perfusate in the recirculating system after a 30-minute equilibration period, serving as an internal control. Group 3: The IPK was treated only with 1 mL of venom fractions of MP (280 μg/mL), added to 100 mL of perfusate in the recirculating system after a 30-minute equilibration period, serving as an internal control. Group 4: The IPK was treated only with 1 mL of venom fractions of LAAO (135 μg/mL), added to 100 mL of perfusate in the recirculating system after a 30-minute equilibration period, serving as an internal control. Group 5: The IPK was treated only with 1 mL of venom fractions of PDE (100 μg/mL), added to 100 mL of perfusate in the recirculating system after a 30-minute equilibration period, serving as an internal control.

The experimental period in each group lasted for 120 minutes of perfusion time for the determination of renal functions, cytokines, and antioxidant oxidative stress parameters in both perfusate and urine samples after envenomation. At the end of the study, portions of the IPK were immediately immersed in liquid nitrogen for further tissue processing to analyze the activities of SOD and CAT, and concentrations of GSH and MDA in the kidney.

### The production of biomarkers for oxidative stress and cytokines

The objective of the present study was to identify biomarkers of oxidative stress and cytokine levels in urine, plasma, and renal tissue. This goal was accomplished by comparing data obtained from in vivo experiments conducted in rabbits and experiments using the IPK model treated with RVV and its venom fractions (PLA_2_, MP, LAAO, and PDE). 

### Determination of oxidative stress parameters

The samples of the left kidney tissue were analyzed for the activities of SOD and CAT, as well as concentrations of reduced GSH and MDA. Additionally, concentrations of both reduced GSH and MDA were determined in the samples of urine, plasma, and perfusate.

*Determination of renal catalase activity (CAT)*

The catalase activity (CAT) of the left kidney tissue sample was determined using the UV spectrophotometric method, as described by a modified version of the method presented in references [26, 27]. In brief, one gram of frozen left kidney tissue was homogenized in a glass homogenizer containing 1% Triton X-100 at 4 °C. This homogenate was then centrifuged at 12,000 rpm (9800 g) for 10 minutes, and the resulting supernatant was collected to assess CAT activity. To assay CAT activity, 0.1 mL of the supernatant was mixed with 1.9 mL of phosphate buffer and 1 mL of 30 mM H_2_O_2_ as a substrate. The UV absorption of the H_2_O_2_ solution was recorded at 240 nm after the H_2_O_2_ had reacted with catalase, utilizing the molar extinction coefficient of H_2_O_2_, every 15 seconds for three minutes. The enzyme activity of the homogenized kidney cortex was calculated based on the decrease in optical density, using enzyme catalase as an external standard. Protein concentration in the supernatant was determined using a Bradford assay. The results were expressed as unit catalase/mg protein.

*Determination of renal superoxide dismutase activity (SOD)*

Superoxide dismutase activities (total SOD) were determined using a previously described method [28] with modification. Briefly, one gram of frozen left kidney tissue was homogenized in a glass homogenizer containing 0.05 M phosphate buffer, pH 7.8, on ice and cleared by centrifugation at 10,000 rpm for 10 minutes at 4 °C. The resulting supernatant was collected to determine SOD activity. For the SOD assay, the reaction volume contained 2.9 mL of xanthine solution (5 µmol xanthine in 10 mL 0.001 N NaOH) and 50 µL of the tissue supernatant, which was mixed to react with 50 µL of xanthine oxidase in 0.1 mM EDTA. Measurements were taken at 550 nm every 30 seconds for four minutes using a visible spectrophotometer (DU650 spectrophotometer, Beckman Coulter ^TM^, USA). The enzyme activity of SOD was calculated from the decrease in optical density, using superoxide dismutase as an external standard. In the determination of SOD activity, the small amount of protein concentration of the supernatant was determined using a Bradford assay. The results were expressed as units of SOD per milligram of protein.

*Determination of glutathione reductase level (GSH)*

The frozen samples of left kidney tissue, plasma, perfusate, and urine were assessed for glutathione reductase levels (GSH) using a modified method [29, 30]. In brief, one gram of frozen left kidney tissue was homogenized in a glass homogenizer containing 0.1 M phosphate buffer at pH 7.4 on ice and centrifuged at 12,000 rpm for 15 minutes at 4 °C. The supernatant was collected for the determination of GSH levels. GSH concentrations in the samples were determined by deproteinizing 100 µL of each sample (tissue supernatant, frozen plasma, frozen perfusate, and frozen urine) with 100 µL of 20% TCA in 1 mM EDTA for five minutes at room temperature. All solution samples were then centrifuged at 2000 rpm for 10 minutes, and each supernatant was collected. The reaction volume contained 200 µL of the supernatant and 1.8 mL of a solution (0.1 mM DTNB, 0.1 PBS, 1% sodium citrate). Optical density measurements were taken at 412 nm using a visible spectrophotometer (DU650 spectrophotometer, Beckman Coulter ^TM^, USA) for the determination of GSH concentration. The protein concentration of the supernatant sample was determined using a Bradford assay. The result of GSH concentration in kidney tissue was expressed as µmol/mg of protein.

*Determination of lipid peroxidation*

Lipid peroxide formation was assessed by measuring thiobarbituric acid-reacting substances (TBARS) in the samples. The method involves the reaction of one molecule of malondialdehyde and two molecules of TBA to form a red malondialdehyde-TBA complex, which can be quantified spectrophotometrically at 532 nm. Malondialdehyde (MDA) in the samples (plasma, perfusate, urine, and kidney tissue) was determined as an indicator of lipid peroxidation by the substances reactive to thiobarbituric acid-TBARS (thiobarbituric acid-reactive substances) using a modified method [31]. In brief, one gram of frozen left kidney tissue was homogenized in a glass homogenizer containing 9 mL of Tris-HCl buffer with 180 mM KCl, 10 mM EDTA, and 0.02% butylated hydroxytoluene (BHT) at pH 7.4 on ice and cleared by centrifugation at 10,000 rpm for 10 minutes at 4 °C. The supernatant was collected for the determination of MDA levels and the protein concentration of the tissue supernatant. The MDA concentration was determined by adding 100 µl of each sample (tissue supernatant, frozen plasma, frozen perfusate, and frozen urine) to 2 mL of 0.37% TBA in 0.25 N HCl and 15% TCA solution, and allowed to react in boiling water (90 °C) for 10 minutes. After cooling with cold water, the mixtures were centrifuged at 9800 g for 10 minutes at 4 °C, and the supernatant was collected to measure absorbance at 532 nm. The protein concentration of the tissue supernatant was determined using a Bradford assay. The result of MDA concentration in kidney tissue was expressed as nmol/mg of protein. The content of lipid peroxide is expressed in terms of nmol of MDA/gram of wet weight, and the total protein is determined by the Lowry method [32] to correct the MDA level, which is expressed in terms of nmol/mg of protein.

### Quantification of cytokine levels

The plasma, perfusate, and urine samples from rabbits in *in vivo* and IPK studies, obtained at various times after administration with either RVV or venom fractions (PLA_2_, MP, LAAO, and PDE), were utilized for measuring anti- and pro-inflammatory cytokines. In brief, quantitative measurements of rabbit cytokines were conducted on commercial ELISA kits following the manufacturer’s instructions (Nori® Rabbit cytokine ELISA Kit, Genorise Scientific, Inc., USA). The cytokine standards and duplicate tested samples were added into a 96-well microplate pre-coated with a specific antibody against each cytokine. After one hour of incubation at room temperature and three rounds of washing, the detection antibody was added and incubated for one hour at room temperature. Subsequently, the plate was washed three times and the plate was incubated for 20 minutes with biotin-streptavidin Horseradish Peroxidase (HRP) conjugate. After a final wash, the reaction was developed by adding the substrate solution. Within 20 minutes, the reaction was stopped, and the optical densities were measured at 450 nm using a microplate reader (Sunrise, TECAN, Austria). For each group, the levels of anti-inflammatory cytokines (IL-4, IL-5, and IL-10) and pro-inflammatory cytokines (IFN-γ, IL-1β, and TNF-α) were estimated in which the standard curve was constructed using the standard cytokine provided by the detection kit. Various concentrations of the standard cytokines were measured in parallel with the samples. Subsequently, the curve depicting these concentrations and OD values was plotted as the standard curve, and the results were expressed in picograms per milliliter (pg/mL). 

### Calculation for renal functions involving oxidative stress and inflammatory cytokine parameters both in intact kidney and IPK

The GFR and effective renal plasma flow (ERPF) were determined using inulin and PAH clearance, respectively. The procedures and calculations for renal clearance and renal blood flow (RBF) were performed as previously described [5].

Renal vascular resistance (RVR) for an intact kidney was calculated from mean arterial blood pressure and renal blood flow (BP/RBF) using the standard formula as previously described [5]. RVR for the IPK was calculated from the flow rate of perfusate to the kidney and perfusion pressure of the system (PP/perfusate flow rate) using standard techniques as previously described [24].

The percentage of fractional excretions (FE) of mediators was determined using the formula: (UC x Pin / Uin x PC) × 100, where C represents the biomarker concentration for cytokines (TNF-α, IL-1β, IFN-γ, IL-4, IL-5, and IL-10) and oxidative stress (GSH, MDA) in plasma/perfusate (P) and urine (U), while Pin and Uin refer to the inulin concentrations in plasma/perfusate (P) and urine (U), respectively.

### Statistical analysis

The data are presented as mean ± SEM. Significant differences between the internal control and each specified time point of each experimental group were analyzed using one-way ANOVA followed by Bonferroni’s post hoc test, where appropriate, with a p-value < 0.05 considered statistically significant. In addition, Student’s unpaired t-test was used to compare the effects of treatments with RVV or its fraction on the activity of CAT and SOD, or the concentration of GSH and MDA in renal tissues during either *in vivo* or *ex vivo* studies. All data were analyzed by GraphPad Prism 5 for Windows (GraphPad Software, San Diego, CA, USA).

## Results

### The effects of RVV and venom fractions of PLA_2_, MP, LAAO, and PDE on changes in renal hemodynamics *in vivo* and isolated perfused kidney studies

**Figure 1.**
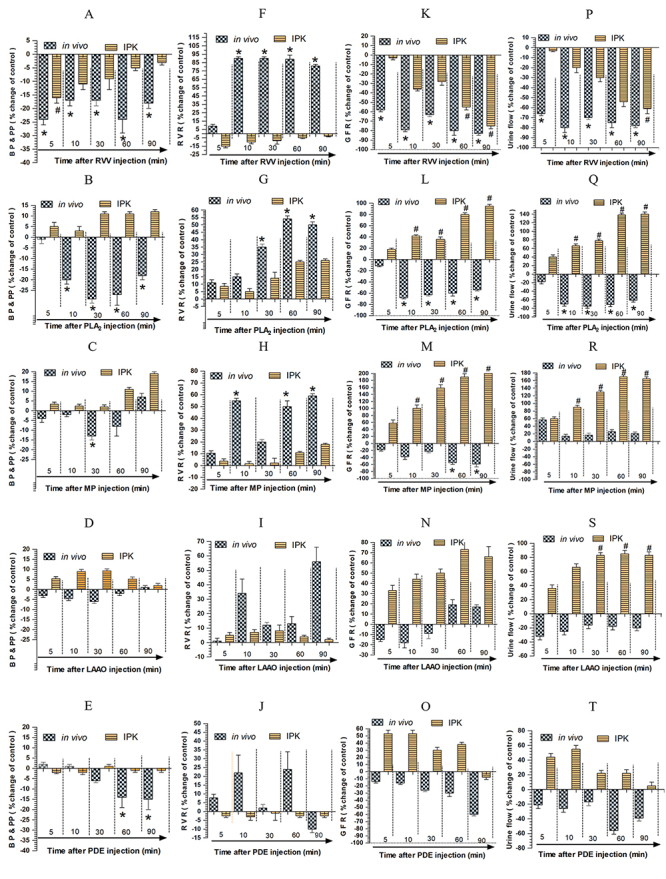
Comparative changes in the percentage responses between arterial blood pressure (BP) *in vivo* and perfusion pressure (PP) in the IPK model at various time points after administrations of **(A)** RVV, **(B)** PLA_2_, **(C)** MP, **(D)** LAAO, and **(E)** PDE. Changes in the percentage of renal vascular resistance (RVR) between *in vivo* and the IPK model at various time points after administrations of (**F**) RVV, (**G**) PLA_2_, (**H**) MP, (**I**) LAAO, and (**J**) PDE. Moreover, changes in the percentage of glomerular filtration rate (GFR) between *in vivo* and the IPK model at various time points after administrations of (**K**) RVV, (**L**) PLA_2_, (**M**) MP, (**N**) LAAO, and (**O**) PDE, as well as changes in the percentage of urine flow at various time points after administrations of (**P**) RVV, (**Q**) PLA_2_, (**R**) MP, (**S**) LAAO, and (**T**) PDE. Each treated group's data were expressed as mean ± SEM, n = 4. A significant difference is denoted by #: p < 0.05 for IPK; *: p < 0.05 for *in vivo*; using repeated measures ANOVA with Bonferroni post-hoc test between the value at the specified time point and the internal control in the same group.

Renal hemodynamics are presented in Figure 1. Based on these results, a series of experiments were conducted to determine the effect of administering RVV or venom fractions of PLA_2_, MP, LAAO, and PDE on renal functions in rabbits, both *in vivo* and IPK groups (Figures 1A to 1T).

*Effects of RVV and its venom fractions on blood pressure (BP) and perfusion pressure (PP)*

The administration of RVV, either *in viv*o (0.1 mg/kg, i.v.) or in the IPK model (1 mg/100 mL perfusate), revealed a consistent decrease in both BP and PP below the control level throughout the experimental periods, as observed in both the *in vivo* (p < 0.05) and IPK model groups (Figure 1A). The injection of PLA_2_ in rabbits (0.2 mg/kg, i.v.) caused a significant reduction in BP (p < 0.05) starting at 10 minutes, ranging from 18% to 30% below the control level throughout the experimental periods after venom injection. However, in the IPK model group, the administration of PLA_2_ (280 µg/100 mL perfusate) consistently caused an increase in PP, ranging from 4% to 12% above the control level throughout the experimental periods (Figure 1B). The effects of MP injection *in vivo* (0.2 mg/kg, i.v.) on the BP of rabbits were shown to significantly decrease below the control level 60 minutes after injection. However, in the IPK model groups, the administration of MP (280 µg/100 mL perfusate) consistently caused an increase in PP, ranging from 4% to 12% above the control level throughout the experimental periods (Figure 1C). The injection of LAAO in rabbits (0.15 mg/kg, i.v.) showed a slight decrease in BP in the *in vivo* group, whereas the administration of LAAO (135 µg/100 mL perfusate) in the IPK model exhibited slight increases in PP throughout the experimental periods (Figure 1D). The effect of PDE injection *in vivo* (0.1 mg/kg, i.v.) on BP showed a significant decrease below the control level at 60 and 90 minutes (p < 0.05) after PDE injection, whereas the administration of PDE (100 µg/100 mL perfusate) in the IPK model showed slight reductions in PP throughout the experimental period (Figure 1E).

*Effects of RVV and its venom fractions on renal vascular resistances (RVR)*

 The administration of RVV in the *in vivo* group (0.1 mg/kg, i.v.) significantly increased RVR (p < 0.05) above the control level, while progressively decreasing it below the control level in the IPK model (1 mg/100 mL perfusate) throughout the experimental periods (Figure 1F). The injection of PLA_2_ led to increased RVR in both the *in vivo* rabbits (0.2 mg/kg, i.v.) and the IPK model groups (280 µg/100 mL perfusate). The increases ranged from 12% to 55% above the control level, with more significance (p < 0.05) occurring at 30 to 90 minutes post-PLA_2_ injection in the *in vivo* study (Figure 1G). The injection of MP *in vivo* (0.2 mg/kg, i.v.) and the IPK model groups (280 µg/100 mL perfusate) increased RVR in both *in vivo* and the IPK model, with a more pronounced increase of 10-60% above the control level at 10, 60, and 90 minutes post-MP injection in the *in vivo* study (Figure 1H). The injection of LAAO in rabbits showed slightly increased RVR in both the *in vivo* (0.15 mg/kg, i.v.) and the IPK model (135 µg/100 mL perfusate) post-MP injection throughout the experimental periods (Figure 1I). The injection of PDE *in vivo* (0.1 mg/kg, i.v.) showed non-significant increases at 10 and 60 minutes after PDE injection, while slight decreases in RVR were apparent in the IPK model after the administration of PDE (100 µg/100 mL perfusate) (Figure 1J).

*Effects of RVV and its venom fractions on glomerular filtration rate (GFR)*

Figure 1K illustrates significant and progressive decreases (p < 0.05) in GFR post-RVV administration in both the *in vivo* model (0.1 mg/kg, i.v.) and IPK model (1 mg/100 mL perfusate) throughout the experimental studies. Moreover, the decrease in GFR was more pronounced at 60 and 90 minutes post-RVV administration in the IPK model. Figure 1L illustrates significant and progressive decreases in GFR (p < 0.05) starting at 10 to 90 minutes post-PLA_2_ injection (0.2 mg/kg, i.v.) in the *in vivo* model. Conversely, administration of PLA_2_ in the IPK model (280 µg/100 mL perfusate) exhibited a significant increase in GFR, progressing from 10 to 90 minutes (p < 0.05). As illustrated in Figure 1M, significant progressive decreases in GFR were observed at 60 and 90 minutes (p < 0.05) post-MP injection in the *in vivo* model (0.2 mg/kg, i.v.). In contrast, post-MP administration in the IPK model groups (280 µg/100 mL perfusate) showed significant progressive increases (p < 0.05) in GFR throughout the experimental periods. In Figure 1N, post-LAAO injection in the *in vivo* model (0.15 mg/kg, i.v.) showed non-significant decreases in GFR in the first 30 minutes, followed by a slight increase at 60-90 minutes. In the IPK model, non-significant increases in GFR were observed post-LAAO administration (135 µg/100 mL perfusate) throughout the experimental period. As illustrated in Figure 1O, there were non-significant, progressive decreases in GFR observed as percent changes of the control after PDE injection in the *in vivo* studies (0.1 mg/kg, i.v.). In contrast, there were non-significant increases in GFR after PDE administration (100 µg/100 mL perfusate) in the IPK model ***.***

*Effects of RVV and its venom fractions on the rate of urine flow*

After RVV injection (0.1 mg/kg, i.v.), urine flow exhibited a marked decrease (p < 0.05) in the intact kidney throughout the experimental periods. Meanwhile, post-RVV administration in the IPK model (1 mg/100 mL perfusate) showed a more prominent decrease (p < 0.05) at the 90-minute mark of the experimental period (Figure 1P). Following PLA_2_ injection (0.2 mg/kg, i.v.), urine flow exhibited a marked decrease (p < 0.05) throughout the experimental period in the *in vivo* group. In contrast, the IPK group demonstrated a significant increase in urine flow (p < 0.05) throughout the 90 minutes after PLA_2_ administration (280 µg/100 mL perfusate) (Figure 1Q). A slight increase in urine flow was observed *in vivo* after MP injection (0.2 mg/kg, i.v.), whereas urine flow in the IPK model significantly increased (p < 0.05) after MP administration (280 µg/100 mL perfusate) throughout the 90-minute experimental period (Figure 1R). Significant increases in urine flow (p < 0.05) were observed in the IPK model at 30, 60, and 90 minutes post-LAAO administration (135 µg/100 mL perfusate), while urine flow slightly decreased after LAAO injection in the *in vivo* model (0.15 mg/kg, i.v.) throughout the experimental period (Figure 1S). As illustrated in Figure 1T, there were non-significant reductions in urine flow after PDE injection (0.10 mg/kg, i.v.) in the *in vivo* studies. In contrast, after PDE administration (100 µg/100 mL perfusate) in the IPK model, non-significant increases in urine flow were observed throughout the experimental period. 

### The effects of RVV and venom fractions (PLA_2_, MP, LAAO, and PDE) on the oxidative stress status of the kidney were assessed both *in vivo* and in IPK

*The effects of RVV or venom fractions administration on the SOD and CAT activity in the kidney in vivo and IPK*

**Figure 2.**
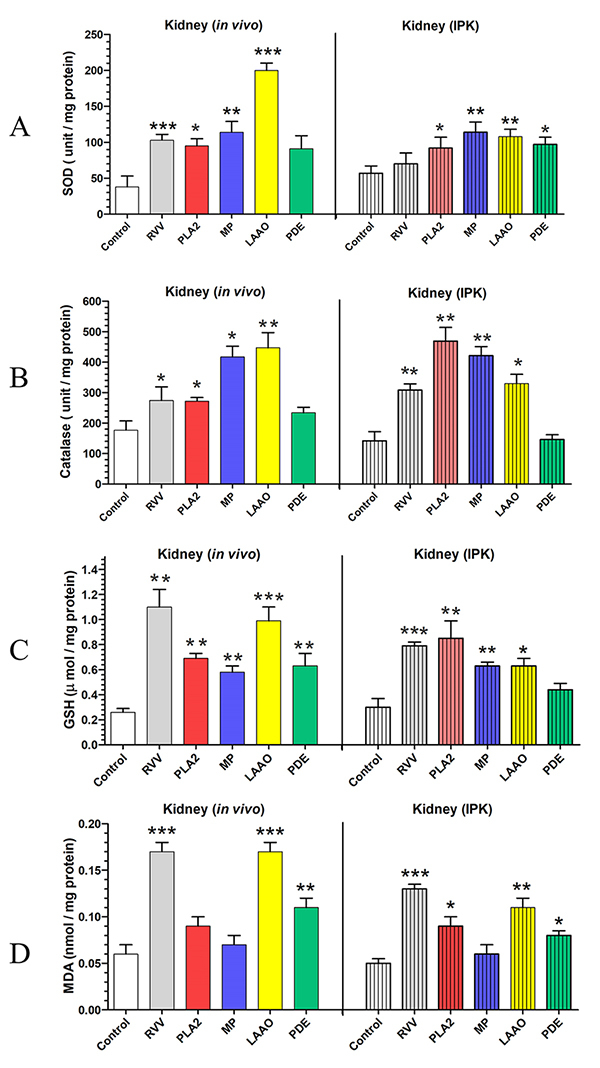
Comparative changes in oxidative stress parameters between the rabbit kidney *in vivo* and the IPK model, including **(A)** the activity of superoxide dismutase (SOD), **(B)** the activity of catalase (CAT), **(C)** the concentration of glutathione-S-transferase (GSH), and **(D)** the concentration of malondialdehyde (MDA), in response to administrations of RVV, PLA_2_, MP, LAAO, and PDE in either the rabbit kidney *in vivo* or the rabbit IPK. Group data were expressed as mean ± SEM, n = 4. *: p < 0.05, **: p < 0.01, ***: p < 0.001, compared with the control group, using an unpaired t-test.

In *in vivo* studies, injections of RVV (0.1 mg/kg, i.v.), PLA_2_ (0.2 mg/kg, i.v.), MP (0.2 mg/kg, i.v.), and LAAO (0.15 mg/kg, i.v.) caused marked increases in SOD activities in the kidney tissues compared with the control group by +171% (p < 0.001), +147% (p < 0.05), +200% (p < 0.01), and +426% (p < 0.001), respectively. Additionally, the treatment of PDE (0.10 mg/kg, i.v.) showed a non-significant increase in SOD activity compared with the control intact kidney (Figure 2A, *in vivo*). For the rabbit IPK administered with RVV (1 mg/100 mL perfusate), there was a non-significant increase (+23%) in SOD activity in the kidney tissue compared with the control rabbit IPK, while administrations of PLA_2_ (280 µg/100 mL perfusate) and MP (280 µg/100 mL perfusate) showed significant increases in SOD activity by +61% (p < 0.05) and +100% (p < 0.01), respectively. The administration of LAAO (135 µg/100 mL perfusate) significantly raised SOD activity in the IPK model by +89% (p < 0.01), while the treatment of PDE (100 µg/100 mL perfusate) showed a significant increase in SOD activity (+70%, p < 0.05) (Figure 2A, IPK). The rabbits treated with RVV and venom fractions showed that CAT activity in kidney tissue increased after injection in the RVV group (0.1 mg/kg, i.v.) by +55% (p < 0.05), in the PLA_2_ group (0.2 mg/kg, i.v.) by +54% (p < 0.05), and in the MP group (0.2 mg/kg, i.v.) by +135% (p < 0.05). Administration of LAAO (0.15 mg/kg, i.v.) showed a marked increase in CAT activity by +152% (p < 0.01), while the injection of PDE (0.10 mg/kg, i.v.) resulted in a non-significant increase in CAT activity (+32%) compared to the control intact kidney (Figure 2B, *in vivo*). The rabbit IPK treated with RVV and venom fractions showed a similar pattern of response for the level of CAT activity as those treated in vivo. Administrations of RVV (1 mg/100 mL perfusate), PLA_2_ (280 µg/100 mL perfusate), MP (280 µg/100 mL perfusate), and LAAO (135 µg/100 mL perfusate) significantly increased CAT activity in the IPK by +117% (p < 0.01), +230% (p < 0.01), +196% (p < 0.01), and +132% (p < 0.05), respectively, while the treatment of PDE (100 µg/100 mL perfusate) showed a slight increase in CAT activity (+3%) in the rabbit IPK (Figure 2B, IPK).

*Effects of RVV or venom fraction administration on the concentrations of GSH and MDA in the kidney in vivo and IPK*

RVV injection in rabbits (0.1 mg/kg, i.v.) significantly increased the concentration of GSH in the kidney tissue by +323% (p < 0.01). Venom fractions caused increases in the concentrations of GSH in the kidney tissue after injection of PLA_2_ (0.2 mg/kg, i.v.) by +165% (p < 0.01) and of MP (0.2 mg/kg, i.v.) by +123% (p < 0.01). Injection of LAAO (0.15 mg/kg, i.v.) caused a marked increase in the concentration of GSH in the kidney tissue by +281% (p < 0.001), and injection of PDE (0.10 mg/kg, i.v.) showed a significant increase in the concentration of GSH by +142% (p < 0.01) compared with the control intact kidney (Figure 2C, *in vivo*). Administration of RVV (1 mg/100 mL perfusate) in the IPK model caused a significant increase in the concentration of GSH in the kidney tissue by +163% (p < 0.001). Administration of venom fractions for PLA_2_ (280 µg/100 mL perfusate), MP (280 µg/100 mL perfusate), and LAAO (135 µg/100 mL perfusate) significantly increased the concentration of GSH in the kidney tissue by +183% (p < 0.01), +110% (p < 0.01), and +110% (p < 0.05), respectively, while the administration of PDE (100 µg/100 mL perfusate) showed a non-significant increase in the concentration of GSH (+47%) in the rabbit IPK compared to the control IPK group (Figure 2C, IPK).

Injection of RVV and venom fractions on the concentration of MDA in the kidney tissue showed some differences (Figure 2D, *in vivo*). Injection of RVV (0.1 mg/kg, i.v.) caused a significant increase in the concentration of MDA in the kidney tissue by +183% (p < 0.001) compared to the control kidney, while injection of both PLA_2_ (0.2 mg/kg, i.v.) and MP (0.2 mg/kg, i.v.) showed non-significant increases in the concentration of MDA in the kidney tissue by +50% and 17%, respectively. Injection of either LAAO (0.15 mg/kg, i.v.) or PDE (0.10 mg/kg, i.v.) caused significant increases in the concentration of MDA in the kidney tissue by +183% (p < 0.001) and +83% (p < 0.01), respectively, compared to the control kidney. The present study demonstrated that the administration of RVV (1 mg/100 mL perfusate) in the IPK model caused a significant increase in the MDA concentration in the kidney tissue by +160% (p < 0.001) compared to the control IPK group (Figure 2D, IPK). Administration of the venom fraction for PLA_2_ (280 µg/100 mL perfusate) also caused a significant increase in the MDA concentration in the kidney tissue by +80% (p < 0.05), whereas administration of MP (280 µg/100 mL perfusate) displayed a non-significant increase in the MDA concentration in the kidney tissue by +20% compared to the control IPK. The administration of LAAO (135 µg/100 mL perfusate) significantly elevated the MDA concentration in the kidney tissue (+120%, p < 0.01), and the administration of PDE (100 µg/100 mL perfusate) also significantly increased the MDA concentration (+60%, p < 0.05) in kidney tissue in the IPK model.

### Effect of RVV and venom fractions administration on the concentration of MDA in urine, plasma/perfusate, and urinary fractional excretion of MDA (FE_MDA_) *in vivo* and IPK studies

**Figure 3.**
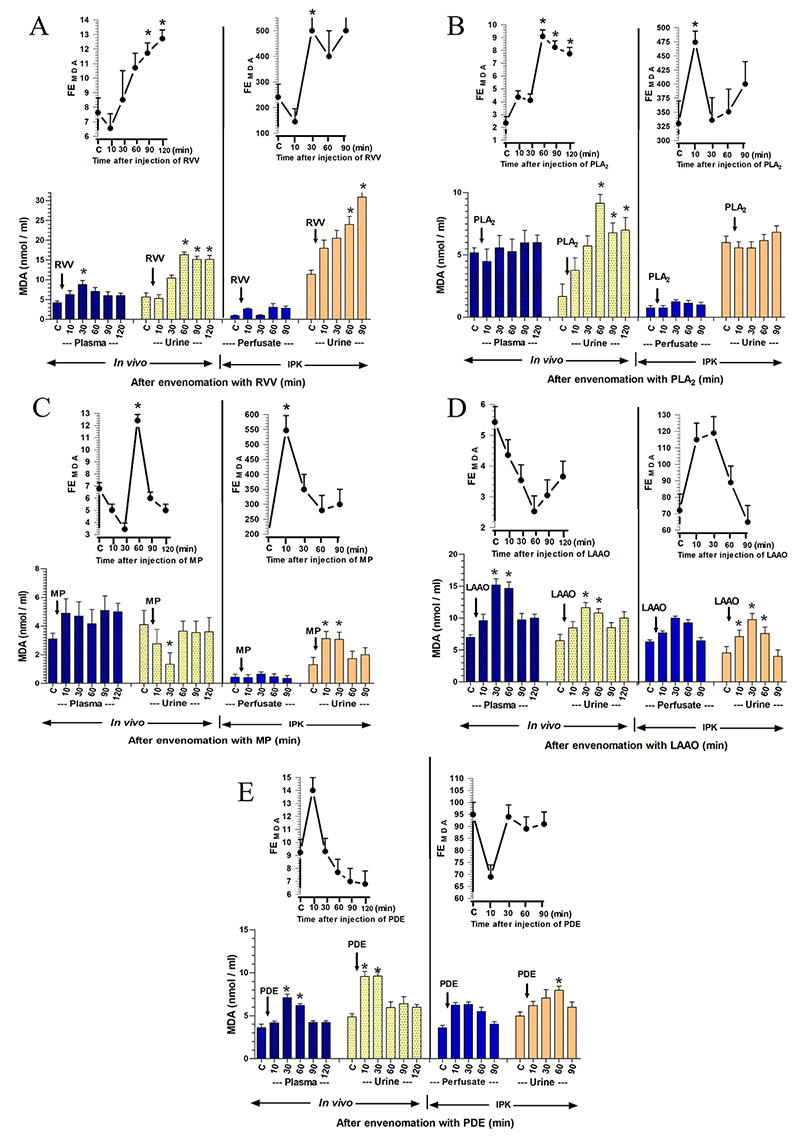
The comparison of two datasets between *in vivo* and rabbit IPK examines the effect of RVV and venom fractions administration on changes in the concentration of MDA in plasma/perfusate, urine, and urinary fractional excretion (FE) at various time points following the administration of **(A)** RVV, **(B)** PLA_2_, **(C)** MP, **(D)** LAAO, and **(E)** PDE. The data are presented as the mean ± SEM (n = 4). *Significant differences (p < 0.05) were analyzed using repeated measures ANOVA with Bonferroni post-hoc test, comparing the specified time point to the internal control within the same group.

The injection of RVV *in vivo* (0.1 mg/kg, i.v.) caused progressive increases in the concentrations of MDA, a marker of lipid peroxidation, both present in plasma and urine after RVV injection (Figure 3A). The concentrations of plasma MDA increased from 4.2 ± 0.4 nmol/mL at time 0 (control) to a maximal response at 30 minutes (8.8 ± 1.0 nmol/mL, p < 0.05), while non-significant increases in the concentrations of MDA were apparent after RVV injection by 7.0 ± 1.0, 6.1 ± 1.0, and 6.0 ± 0.6 nmol/mL at 60, 90, and 120 minutes, respectively, when compared to their respective time 0 values. A similar pattern was observed with concentrations of MDA in urine, with values becoming higher than those seen at control time 0 (5.7 ± 1.0 nmol/mL) at 30 minutes (10.4 ± 0.8), 60 minutes (16.3 ± 0.7, p < 0.05), 90 minutes (15.1 ± 0.8, p < 0.05), and 120 minutes (15.2 ± 1.0 nmol/mL, p < 0.05), respectively. The %FE_MDA_ initially decreased within the first 10 minutes after RVV injection *in vivo* and then increased progressively, with marked effects at 90 and 120 minutes (p < 0.05) post-injection. In the IPK model, administration of RVV (1 mg/100 mL perfusate) showed a slight increase in the levels of MDA in perfusate from 0.9 ± 0.2 nmol/mL at time 0 to 2.6 ± 0.2, 1.0 ± 0.1, 3.0 ± 1.0, and 2.8 ± 0.5 nmol/mL at 10, 30, 60, and 90 minutes, respectively. The MDA concentration in urine increased progressively from 11.4 ± 1.0 nmol/mL at time 0 (control) to a significant effect by 24.0 ± 2.0 at 90 minutes (p < 0.05) and 30.0 ± 4.0 nmol/mL at 120 minutes (p < 0.05) after RVV administration. The percentage of FE_MDA_ in the IPK model initially decreased within the first 10 minutes and then increased progressively with a marked effect at 30 minutes (p < 0.05) after RVV administration.

The effect of PLA_2_ injection (0.2 mg/kg, i.v.) on lipid peroxidation is shown in Figure 3B. The concentrations of MDA in plasma showed no significant changes in the intact kidney, although they initially decreased within the first 10 minutes from 5.4 ± 0.4 nmol/mL at time 0 to 4.4 ± 0.4 nmol/mL at 10 minutes and returned to the control values within the range of 5.5 to 6.0 nmol/mL from 30 to 120 min. The concentrations of MDA in urine displayed progressive increases in the intact kidney and exhibited a significant elevation from 1.7 ± 0.9 nmol/mL at time 0 to higher responses at 60 minutes (9.2 ± 0.7 nmol/mL, p < 0.05), 90 minutes (6.8 ± 0.8 nmol/mL, p < 0.05), and 120 minutes (7.0 ± 1.1 nmol/mL, p < 0.05), respectively, after PLA_2_ injection. The %FE_MDA_ increased progressively after PLA_2_ injection with marked effects at 60, 90, and 120 minutes (p < 0.05) compared to the value at 0-time. In the IPK model, administration of PLA_2_ (280 µg/100 mL perfusate) showed no significant alterations in the levels of MDA concentrations in either the perfusate or urine. The percentage of FE_MDA_ in IPK showed a significant increase (p < 0.05) in the first 10 minutes and decreased afterward to the control level after PLA_2_ administration.

In Figure 3C, injection of MP (0.2 mg/kg, i.v.) *in vivo* increased the plasma MDA concentration from 3.1 ± 0.4 nmol/mL at time 0 to 4.9 ± 1.0 nmol/mL at 10 minutes after MP injection, and it remained higher than the control level throughout the experiment. The concentration of MDA in urine in the intact kidney decreased from 4.1 ± 1.0 nmol/mL at time 0 to 2.8 ± 1.0 nmol/mL at 10 minutes and showed a significant decrease by 1.3 ± 0.8 nmol/mL (p < 0.05) at 30 minutes post-MP injection. Administration of MP (280 µg/100 mL perfusate) in the IPK model showed no significant change in the concentration of MDA in the perfusate from 0.43 ± 0.2 nmol/mL at time 0 to a similar range of concentrations throughout experimental periods after MP administration. The concentrations of MDA in the urine of the IPK significantly increased from 1.3 ± 0.5 nmol/mL at time 0 to 3.1 ± 0.5 and 3.0 ± 0.5 nmol/mL (p < 0.05) at 10 and 30 minutes, respectively, after MP administration. Injection of MP into *in vivo* rabbits caused a triphasic effect on FE_MDA_ consisting of an initial transient reduction that occurred within the first 30 minutes, followed by an increase that peaked at 60 minutes (p < 0.05) after MP injection. After, a terminal reduction was usually observed that started between 90 and lasted until 120 minutes before the end of the experiment. The percentage of FE_MDA_ in the IPK model significantly increased at 60 minutes (p < 0.05) post-MP injection and then decreased to the control level thereafter.

The effect of LAAO on lipid peroxidation is illustrated in Figure 3D. *In vivo,* injection of LAAO (0.15 mg/kg, i.v.) revealed a consistent increase in the concentration of MDA in both plasma and urine throughout the experimental period following LAAO administration. In plasma, the MDA concentration became significant, escalating from 7.0 ± 0.4 nmol/mL at time 0 to 15.2 ± 1.0 and 14.7 ± 1.2 nmol/mL (p < 0.05) at 30 and 60 minutes post-LAAO injection, respectively. Simultaneously, the MDA concentration in urine exhibited a significant increase, rising from 6.5 ± 1.0 nmol/mL at time 0 to 11.6 ± 0.8 and 10.8 ± 0.7 nmol/mL (p < 0.05) at 30 and 60 minutes post-LAAO injection, respectively. In the IPK model, the administration of LAAO (135 µg/100 mL perfusate) also induced increases in the concentrations of MDA in perfusate and urine. The MDA concentration in plasma increased from 6.3 ± 0.37 nmol/mL at time 0 to a peak of 10.3 ± 0.3 nmol/mL at 30 minutes post-LAAO administration. Notably, the MDA concentration in urine showed a significant increase, starting from 6.3 nmol/mL at time 0 to 7.1 ± 1.0, 9.7 ± 1.0, and 8.0 ± 1.0 nmol/mL (p < 0.05) at 10, 30, and 60 minutes post-LAAO administration, respectively. The percentage of FE_MDA_ in the intact kidney gradually decreased, reaching its lowest level at 60 minutes after LAAO injection, and subsequently exhibited a slow increase towards the control level. Conversely, in the IPK model, the percentage of FE_MDA_ increased, reaching its peak at 30 minutes after LAAO administration, and then decreased to the control level thereafter.

The effect of the PDE fraction on lipid peroxidation is presented in Figure 3E. The concentration of MDA in both plasma and urine exhibited a consistent increase throughout the experimental period. Injection of PDE (0.10 mg/kg, i.v.) *in vivo* demonstrated a significant rise in the concentration of MDA in plasma from 3.6 ± 0.4 nmol/mL at time 0 to a peak of 7.1 ± 0.4 and 6.2 ± 0.2 nmol/mL at 30 and 60 minutes (p < 0.05) after PDE injection, respectively. The concentration of MDA in urine significantly increased from 4.8 ± 0.4 nmol/mL at time 0 to a peak of 9.6 ± 0.6 nmol/mL at both 30 and 60 minutes (p < 0.05) after PDE injection. In the IPK model, administration of PDE (100 µg/100 mL perfusate) resulted in increases in the concentration of MDA in both perfusate and urine displaying a continuous increase during the experimental period. The concentration of MDA in perfusate increased from 3.6 ± 0.3 nmol/mL at time 0 to a peak of 6.2 ± 0.3 and 6.3 ± 0.3 nmol/mL at 10 and 30 minutes, respectively, while a significant increase in MDA concentration in urine observed from 4.9 ± 0.5 nmol/mL at time 0 to a peak of 7.9 ± 0.5 nmol/mL at 60 minutes (p < 0.05) after PDE administration. The percentage of FE_MDA_ in the intact kidney showed an initial increase within 10 minutes after the PDE injection and gradually decreased to reach the control level thereafter. On the other hand, the percentage of FE_MDA_ in the IPK model decreased within 10 minutes after PDE injection and subsequently returned near the control level.

### Effect of RVV and venom fraction administration on the concentration of glutathione reductase (GSH) in urine, plasma/perfusate, and urinary fractional excretion of GSH (FE_GSH_) *in vivo* and IPK studies

**Figure 4.**
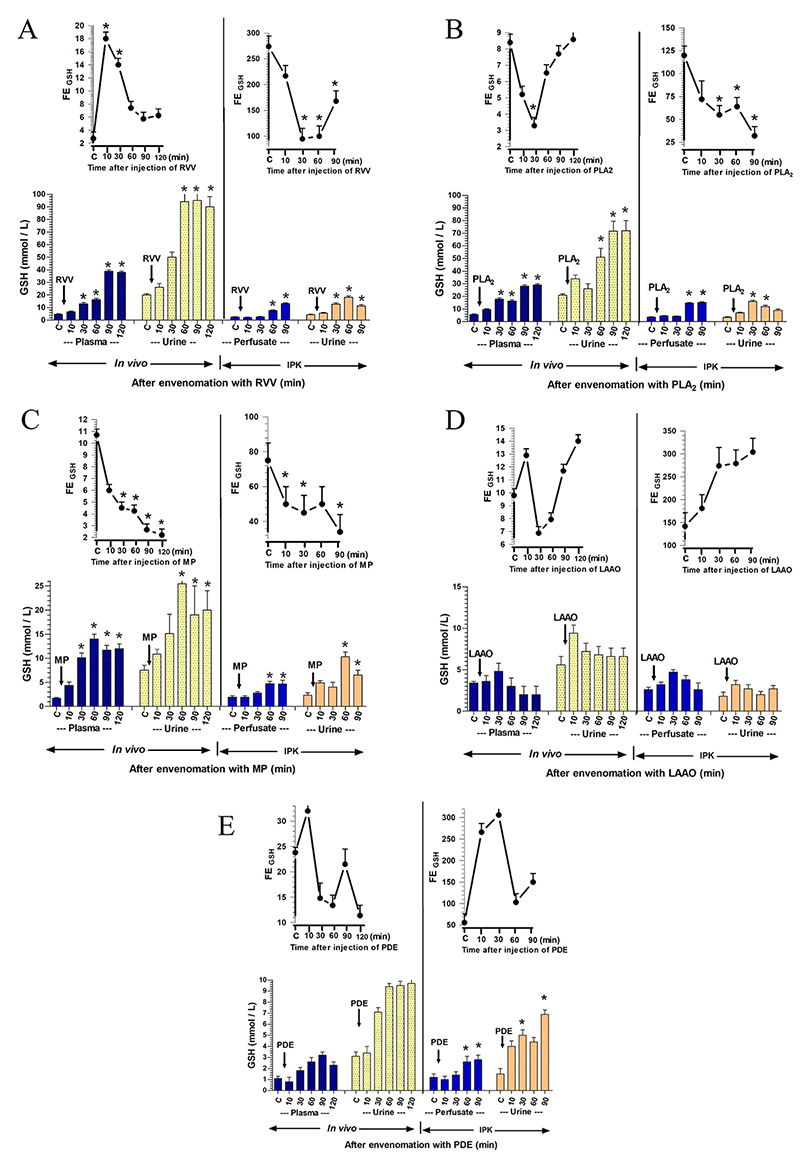
The comparison of two datasets between *in vivo* and rabbit IPK examines the effect of RVV and venom fractions administration on changes in the concentration of GSH in plasma/perfusate, urine, and urinary fractional excretion (FE) at various time points following the administration of **(A)** RVV, **(B)** PLA_2_, **(C)** MP, **(D)** LAAO, and **(E)** PDE. The data are presented as the mean ± SEM (n = 4). *Significant differences (p < 0.05) were analyzed using repeated measures ANOVA with Bonferroni post-hoc test, comparing the specified time point to the internal control within the same group.

The effect of a single injection of crude RVV (0.1mg/kg, i.v.) on the concentration of GSH, an antioxidant marker, is shown in Figure 4A. GSH concentrations exhibited a significant and progressive increase in both urine and plasma throughout the experimental period in both *in vivo* and IPK models after RVV injection. GSH concentrations in plasma significantly rose from 4.7 ± 0.4 mmol/mL at time 0 to peaks of 38.8 ± 1.0 and 38.0 ± 1.0 mmol/mL at 90 and 120 minutes (p < 0.05), respectively, after RVV administration. Similarly, GSH concentrations in urine significantly increased from 20.0 ± 1.0 mmol/mL at time 0 to peaks of 94.0 ± 7.0, 95.8 ± 8.0, and 90.0 ± 6.0 mmol/mL at 60, 90, and 120 minutes (p < 0.05), respectively, following RVV injection *in vivo*. In the IPK model, the administration of RVV (1 mg/100 mL perfusate) resulted in increases in GSH concentration in perfusate, rising from 2.3 ± 0.3 mmol/mL at time 0 to peaks of 7.7 ± 0.5 and 13.1 ± 0.8 mmol/mL at 60 and 90 minutes (p < 0.05), respectively. Additionally, a significant increase in GSH concentration in urine was observed, starting from 4.3 ± 0.5 mmol/mL at time 0 to 12.7 ± 1.0, 18.2 ± 1.0, and 11.3 ± 1.0 mmol/mL at 30, 60, and 90 minutes (p < 0.05), respectively, after RVV administration. The percentage of FE_GSH_ significantly increased in the intact kidney at 10 and 30 minutes (p < 0.05), subsequently decreasing near the control level. Conversely, in the IPK model, the percentage of FE_GSH_ decreased following RVV administration, reaching its lowest values at 30, 60, and 90 minutes (p < 0.05) compared to the control level.

The effect of PLA_2_ injection (0.2 mg/kg, i.v.) on GSH concentration is illustrated in Figure 4B. The concentrations of GSH in plasma demonstrated a significant increase, rising from 5.6 ± 0.4 mmol/mL at time 0 to a peak of 28.0 ± 1.0 and 29.0 ± 1.0 mmol/mL at 90 and 120 minutes (p < 0.05), respectively, after PLA_2_ injection. Simultaneously, the concentration of GSH in urine progressively increased from 21.0 ± 1.0 mmol/mL at time 0 to 51.0 ± 7.0, 71.5 ± 8.0, and 72.0 ± 8.0 mmol/mL at 60, 90, and 120 minutes (p < 0.05), respectively, following PLA_2_ injection. The effect of PLA_2_ administration (280 µg/100 mL perfusate) in IPK exhibited a significant increase in GSH concentrations in perfusate, escalating from 3.6 ± 0.3 mmol/mL at time 0 to a peak of 15.0 ± 0.5 mmol/mL (p < 0.05) at both 90 and 120 minutes, respectively. Concurrently, concentrations of GSH in urine in the IPK model increased from 3.4 ± 0.5 mmol/mL at time 0 to 15.9 ± 1.0 and 12.0 ± 1.0 mmol/mL (p < 0.05) at 30 and 120 minutes, respectively. The percentage of FE_GSH_ in the intact kidney exhibited a significant decrease (p < 0.05) at 30 minutes post-PLA_2_ injection, followed by a gradual increase toward the control level. Conversely, in the IPK model, the percentage of FE_GSH_ significantly decreased (p < 0.05) steadily throughout the experimental period following PLA_2_ injection.

The effect of MP fraction injection (0.2 mg/kg, i.v.) on the concentration of GSH is shown in Figure 4C. The concentrations of GSH in plasma exhibited a significant increase, escalating from 1.7 ± 0.2 mmol/mL at time 0 to 10.1 ± 1.0, 14.0 ± 1.0, 11.7 ± 1.0, and 12.0 ± 1.0 mmol/mL at 30, 60, 90, and 120 minutes (p < 0.05), respectively, after MP injection. Simultaneously, the concentration of GSH in urine progressively increased from 7.6 ± 1.0 mmol/mL at time 0 to 25.5 ± 7.0, 19.0 ± 6.0, and 20.0 ± 4.0 mmol/mL at 60, 90, and 120 minutes (p < 0.05), respectively, after MP injection. The effect of MP administration (280 µg/100 mL perfusate) in IPK exhibited a significant increase in concentrations of GSH in perfusate, rising from 1.9 ± 0.3 mmol/mL at time 0 to a peak of 4.8 ± 0.5 mmol/mL (p < 0.05) at both 90 and 120 minutes, respectively. Concurrently, concentrations of GSH in urine increased from 2.3 ± 0.5 mmol/mL at time 0 to 10.3 ± 1.0 and 6.5 ± 1.0 mmol/mL (p < 0.05) at 60 and 90 minutes, respectively. The percentage of FE_GSH_ in the intact kidney showed a significant decrease (p < 0.05) that began at 30 minutes and continued throughout the end of the experiment post-MP injection. In a similar pattern, the percentage of FE_GSH_ in the IPK model significantly decreased (p < 0.05) steadily, beginning at 10 minutes and throughout the experimental period after MP administration.

The effect of LAAO injection (0.15 mg/kg, i.v.) on the concentration of GSH *in vivo*, as shown in Figure 4D, revealed that GSH concentrations in plasma initially increased within 30 minutes post-LAAO injection, rising from 3.4 ± 0.2 mmol/mL at time 0 to 4.8 ± 1.0 mmol/mL at 30 minutes (p > 0.05), followed by a gradual decrease thereafter. Similarly, GSH concentrations in urine *in vivo* exhibited non-significant increases throughout the experimental period, starting from 5.6 ± 1.0 mmol/mL at time 0 to 9.4 ± 1.0, 7.2 ± 1.0, 6.8 ± 1.0, and 6.6 ± 1.0 mmol/mL at 10, 30, 60, and both 90 and 120 minutes, respectively, after LAAO injection. In the IPK model, the administration of LAAO (135 µg/100 mL perfusate) also demonstrated non-significant increases in GSH concentrations in both perfusate and urine throughout the experimental period. GSH levels in the perfusate increased from 2.6 ± 0.3 mmol/mL at time 0 to 4.7 ± 0.3 and 3.8 ± 0.5 mmol/mL at 30 and 60 minutes (p > 0.05), respectively. The concentrations of GSH in urine showed a tendency to increase from 1.8 ± 0.5 mmol/mL at time 0 to 3.2 ± 0.5 and 2.7 ± 0.5 mmol/mL (p > 0.05) at 10 and 30 minutes, respectively. LAAO injection induced a biphasic effect on FE_GSH_ in the intact kidney, characterized by an initial transient increase within the first 10 minutes, followed by a decrease at 30 minutes after LAAO injection, and subsequently, a gradual increase thereafter. However, in the IPK model, the percentage of FE_GSH_ increased stepwise throughout the experimental period following the LAAO injection.

The effect of PDE injection (0.1 mg/kg, i.v.) on the concentration of GSH is shown in Figure 4E. The concentrations of GSH in plasma showed a non-significant increase from 1.1 ± 0.2 mmol/mL at time 0 to a peak of 3.2 ± 0.3 mmol/mL at 90 minutes after PDE injection, while the concentration of GSH in urine also exhibited a non-progressive increase from 3.1 ± 0.4 mmol/mL at time 0 to 9.4 ± 0.3, 9.5 ± 0.4, and 9.7 ± 0.4 mmol/mL at 60, 90, and 120 minutes, respectively, after PDE injection. The effect of PDE administration (100 µg/100mL perfusate) in IPK revealed a significant increase in concentrations of GSH in perfusate from 1.2 ± 0.3 mmol/mL at time 0 to a peak of 2.6 ± 0.5 and 2.8 ± 0.4 mmol/mL (p < 0.05) at 60 and 90 minutes, respectively. Simultaneously, concentrations of GSH in urine increased from 1.5 ± 0.5 mmol/mL at time 0 to 5.0 ± 0.50, 4.4 ± 0.4, and 6.9 ± 0.4 mmol/mL (p < 0.05) at 30, 60, and 90 minutes, respectively. The percentage of FE_GSH_ in the intact kidney increased in the first 10 minutes after PDE injection and progressively decreased thereafter. Meanwhile, the percentage of FE_GSH_ in the IPK model increased from the first 10 to 30 minutes after PDE injection and subsequently decreased, approaching the control level.

### Effects of RVV or venom fraction administration on the production of inflammatory cytokines in urine, plasma/perfusate, and urinary fractional excretion (FE) *in vivo* and IPK studies

*The effect of RVV on the production of pro-inflammatory and anti-inflammatory cytokines*

**Figure 5.**
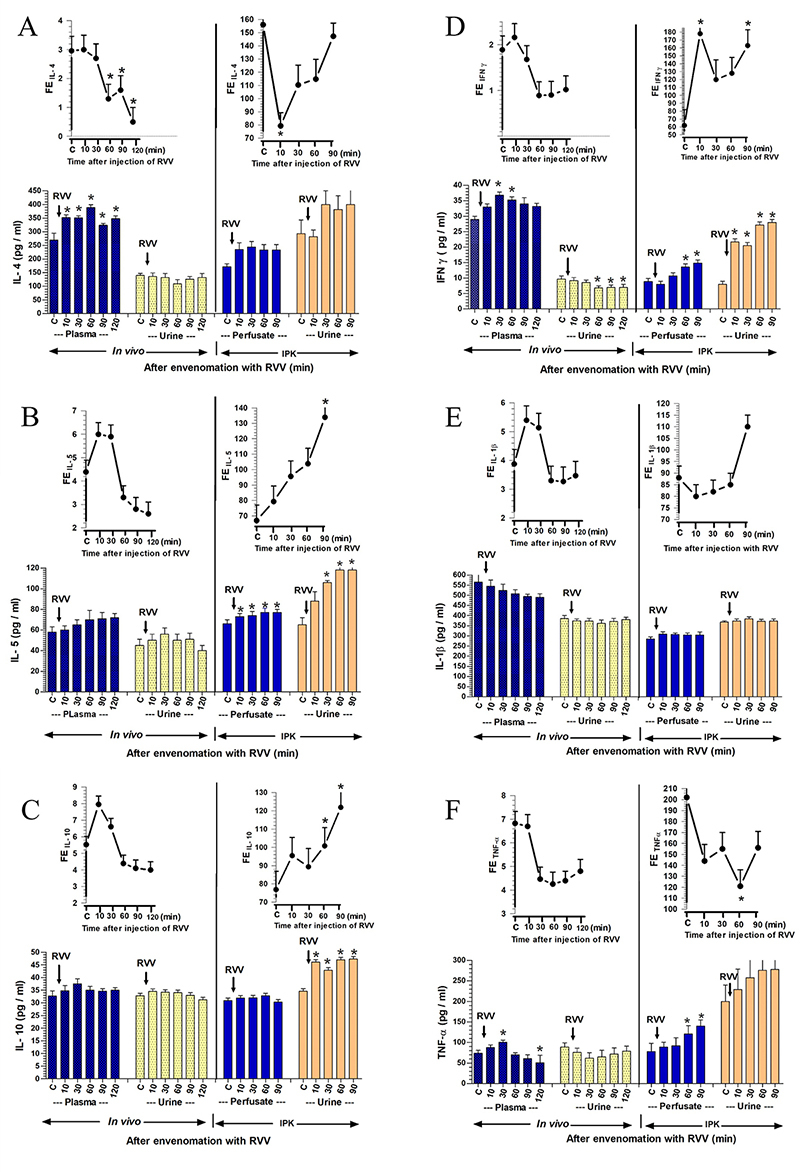
Changes in the concentration of pro-inflammatory and anti-inflammatory cytokines in plasma/perfusate, urine, and urinary fractional excretion (FE) were observed at various time points in response to the administration of RVV in *in vivo* and IPK studies. A comparison of two datasets from the treated groups between *in vivo* and the rabbit IPK after the administrations of RVV was conducted to analyze changes in **(A)** IL-4, **(B)** IL-5, **(C)** IL-10, **(D)** IFN-γ, **(E)** IL-1β, and **(F)** TNF-α. The data are presented as the mean ± SEM (n = 4). *Significant differences (p < 0.05) were determined using repeated measures ANOVA with Bonferroni post-hoc test, comparing the specified time point to the internal control within the same group.

The effects of RVV injection *in vivo* (0.1 mg/kg, i.v.) and in the IPK model (1 mg/100 mL perfusate) on the production of anti-inflammatory and pro-inflammatory cytokines are illustrated in Figure 5. The effect of RVV injection on anti-inflammatory cytokines, particularly IL-4 (Figure 5A), demonstrates that the concentration of IL-4 in plasma showed a significant increase, starting from 270 ± 25 pg/mL at time 0 to 352 ± 10 pg/mL at 10 minutes (p < 0.05) and maintained high significant levels throughout the experimental period. Meanwhile, the concentrations of IL-4 in urine exhibited a trend towards a decrease from 140 ± 8 pg/mL at time 0 to a low value of 109 ± 15 pg/mL at 60 minutes after RVV injection. In the IPK model, the effect of RVV showed non-significant increases in IL-4 concentration in perfusate, starting from 172 ± 11 pg/mL at time 0 to an initial value of 235 ± 25 pg/mL at 10 minutes after RVV administration, while the IL-4 concentration in urine demonstrated non-significant increases from 293 ± 50 pg/mL at time 0 to 400 ± 60 pg/mL at both 30 and 90 minutes after RVV administration. The percentage of FE_IL-4_ significantly decreased (p < 0.05) in the intact kidney at 60, 90, and 120 minutes post-RVV injection. Conversely, in the IPK model, FE_IL-4_ decreased significantly at 10 minutes (p < 0.05) after RVV injection, gradually increasing thereafter, approaching the control level.

The effect of RVV on anti-inflammatory cytokines, specifically IL-5, is shown in Figure 5B. In the *in vivo* study, concentrations of IL-5 in plasma exhibited nonsignificant increases, rising from 58 ± 5 pg/mL at time 0 to a peak of 72 ± 4 pg/mL at 120 minutes, and simultaneously, IL-5 concentrations in urine increased from 45 ± 6 pg/mL at time 0 to a peak of 56 ± 6 pg/mL at 30 minutes after RVV injection. Contrastingly, RVV administration in the IPK model led to significant and progressive increases in IL-5 concentrations in perfusate, starting from 66 ± 4 pg/mL at time 0 to a peak of 77 ± 3 pg/mL (p < 0.05) at 90 minutes, and IL-5 concentrations in urine increased from 65 ± 7 pg/mL at time 0 to a peak of 118 ± 6 pg/mL (p < 0.05) at 90 minutes after RVV administration. The percentage of FE_IL-5_ in the intact kidney increased from the first 10 to 30 minutes after RVV injection and gradually decreased thereafter. Conversely, the percentage of FE_IL-5_ increased stepwise, with significance occurring at 90 minutes post-RVV injection in the IPK model.

The effect of RVV on anti-inflammatory cytokines, specifically IL-10, as illustrated in Figure 5C, revealed nonsignificant increases in IL-10 concentrations in both plasma and urine within the intact kidney model. IL-10 concentrations in plasma slightly increased from 32.7 ± 2 pg/mL at time 0 to 34.5 ± 1 and 37.5 ± 2 pg/mL at 10 and 30 minutes, respectively. Additionally, IL-10 concentrations in urine also exhibited slight increases from 32.8 ± 1 pg/mL at time 0 to 34.5 ± 1 and 34.2 ± 1 pg/mL at 10 and 30 minutes, respectively. In contrast, the concentrations of IL-10 in urine in the IPK model significantly increased from 34 ± 2 pg/mL at time 0 to a peak of 47.3 ± 1 pg/mL (p < 0.05) at 90 minutes, but not in the perfusate, after RVV administration throughout the experimental period. The percentage of FE_IL-10_ in the intact kidney increased in the initial 10 minutes after RVV injection and gradually decreased thereafter, reaching the control level. In the IPK model, the percentage of FE_IL-10_ significantly increased stepwise at 90 minutes (p < 0.05) compared to the control value after RVV injection.

The effect of RVV on pro-inflammatory cytokines, specifically IFN-γ, is shown in Figure 5D. In the *in vivo* study, RVV injection resulted in a significant increase in IFN-γ concentrations in plasma from 29 ± 1 pg/mL at time 0 to 37 ± 1 and 35 ± 1 pg/mL (p < 0.05) at 30 and 60 minutes, respectively. Conversely, concentrations of IFN-γ in urine significantly decreased from 9.7 ± 1 pg/mL at time 0 to 6.8 ± 0.7, 7.0 ± 0.7, and 7.0 ± 0.8 pg/mL (p < 0.05) at 60, 90, and 120 minutes, respectively. In the IPK model, IFN-γ concentrations in perfusate increased from 8.9 ± 1.0 pg/mL at time 0 to 13.6 ± 1.0 and 14.9 ± 1.0 pg/mL (p < 0.05) at 60 and 90 minutes, respectively. IFN-γ concentrations in urine showed significant progressive increases from 8.0 ± 1.0 to 21 ± 1.0 - 28.0 ± 1.0 pg/mL (p < 0.05) at 10-90 minutes after RVV administration. The percentage of FE_IFN-γ_ progressively decreased after RVV injection in the intact kidney model, while in the IPK model, the percentage of FE_IFN-γ_ significantly increased at 10 and 90 minutes (p < 0.05) after RVV administration.

The effect of RVV administration on pro-inflammatory cytokines, particularly IL-1β, is shown in Figure 5E. The *in vivo* study revealed non-significant decreases in the concentrations of IL-1β in plasma, starting from 565 ± 40 pg/mL at 0 minutes to 494 ± 12 pg/mL at 120 minutes post-RVV injection. Likewise, the concentrations of IL-1β in urine exhibited a slight reduction from 385 ± 15 pg/mL at 0 minutes to a nadir of 362 ± 16 pg/mL at 60 minutes post-RVV injection. In the IPK model, the concentrations of IL-1β showed slight increases in perfusate from 285 ± 10 pg/mL at time 0 to 309 ± 12 pg/mL at 10 minutes, and the concentrations of IL-1β in urine slightly increased from 368 ± 6 at time 0 to 384 ± 10 pg/mL at 30 minutes after RVV administration. The percentage of FE_IL-1β_ in the intact kidney increased in the first 30 minutes after RVV injection and gradually decreased after that to the control level. However, in the IPK model, the percentage of FE_IL-1β_ showed no changes within the first 60 minutes and then increased at 90 minutes compared to the control value after RVV administration.

The effect of RVV on pro-inflammatory cytokines for TNF-α is shown in Figure 5F. In the *in vivo* model, concentrations of TNF-α in plasma increased from 74 ± 7 pg/mL at time 0 to 101 ± 5 pg/mL at the first 30 minutes (p < 0.05) and then decreased to 51 ± 18 pg/mL (p < 0.05) at 120 minutes after RVV injection. Concentrations of TNF-α in urine trended towards a decrease from 89 ± 10 pg/mL at time 0 to 62 ± 13, 65 ± 16, 72 ± 15, and 79 ± 12 pg/mL at 30, 60, 90, and 120 minutes, respectively, after RVV injection. In the IPK model, concentrations of TNF-α in perfusate significantly increased from 78 ± 20 pg/mL at time 0 to 121 ± 20 and 140 ± 15 pg/mL (p < 0.05) at 60 and 90 minutes, respectively, post-RVV administration, while concentrations of TNF-α in urine showed non-significant progressive increases from 200 ± 40 pg/mL at time 0 to 276 ± 60 pg/mL at both 60 and 90 minutes after RVV administration. The percentage of FE_TNF-α_ decreased throughout the study period in the intact kidney, while the percentage of FE_TNF-α_ in the IPK models significantly decreased at 60 minutes (p < 0.05) after RVV administration.

*The effect of PLA*_*2*_ *on the production of pro-inflammatory and anti-inflammatory cytokines*

**Figure 6.**
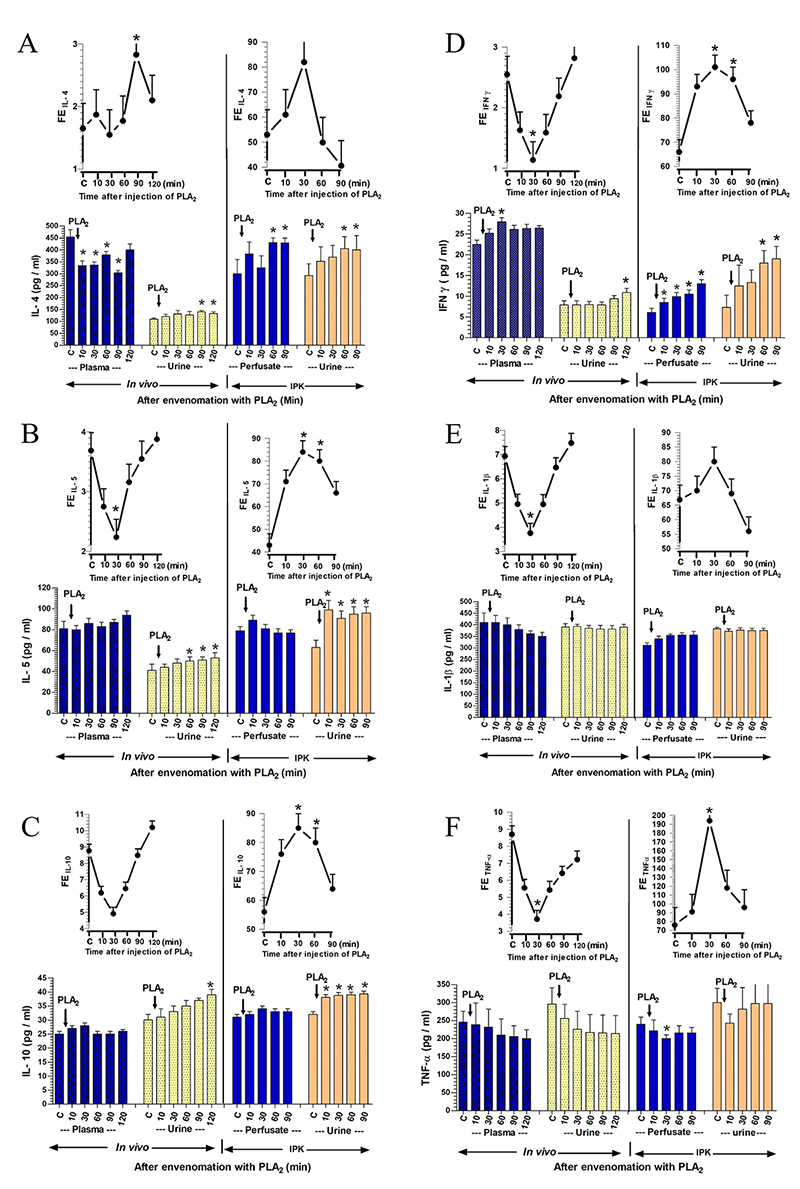
Changes in the concentration of pro-inflammatory and anti-inflammatory cytokines in plasma/perfusate, urine, and urinary fractional excretion (FE) were observed at various time points in response to the administration of PLA_2_ in *in vivo* and IPK studies. A comparison of two datasets from the treated groups between *in vivo* and the rabbit IPK after the administrations of PLA_2_ was conducted to analyze changes in **(A)** IL-4, **(B)** IL-5, **(C)** IL-10, **(D)** IFN-γ, **(E)** IL-1β, and **(F)** TNF-α. The data are presented as the mean ± SEM (n = 4). *Significant differences (p < 0.05) were determined using repeated measures ANOVA with Bonferroni post-hoc test, comparing the specified time point to the internal control within the same group.

The effect of PLA_2_ injection on the production of pro-inflammatory and anti-inflammatory cytokines in plasma and urine is shown in Figure 6. The effects of PLA_2_ injection *in vivo* (0.2 mg/kg, i.v.) and in the IPK model (280 µg/100 mL perfusate) on the production of anti-inflammatory and pro-inflammatory cytokines are illustrated in Figure 6. The effect of PLA_2_ injection in the *in vivo* study on anti-inflammatory cytokines, particularly IL-4 (Figure 6A), demonstrated a significant decrease in IL-4 concentration in plasma, from 455 ± 30 pg/mL at time 0 to 334 ± 20, 337 ± 13, 379 ± 15, and 304 ± 10 pg/mL (p < 0.05) at 10, 30, 60, and 90 minutes, respectively, post-PLA_2_ injection. Contrastingly, the concentrations of IL-4 in urine increased significantly from 109 ± 5 pg/mL at time 0 to higher values of 140 ± 5 and 133 ± 7 pg/mL at 90 and 120 minutes (p < 0.05) after PLA_2_ injection. In the IPK model, the effect of PLA_2_ showed significant increases in IL-4 concentration in perfusate, starting from 300 ± 60 pg/mL at time 0 to 431 ± 20 pg/mL at both 60 and 90 minutes after PLA_2_ administration. The IL-4 concentration in urine demonstrated significant increases from 290 ± 50 pg/mL at time 0 to 406 ± 50 and 400 ± 60 pg/mL (p < 0.05) at both 60 and 90 minutes, respectively, after PLA_2_ administration. The percentage of FE_IL-4_ significantly increased in the intact kidney at 90 minutes (p < 0.05) and decreased thereafter, approaching the control level after PLA_2_ injection. In the IPK model, FE_IL-4_ increased to a higher level at 30 minutes and gradually decreased thereafter, approaching the control level after PLA_2_ administration.

The effect of PLA_2_ on anti-inflammatory cytokines, specifically IL-5, is shown in Figure 6B. In the *in vivo* study, concentrations of IL-5 in plasma exhibited nonsignificant increases, rising from 81 ± 7 pg/mL at time 0 to a peak of 94 ± 4 pg/mL at 120 minutes, and simultaneously, IL-5 concentrations in urine significantly increased from 41 ± 6 pg/mL at time 0 to 50 ± 4, 51 ± 3, and 53 ± 5 pg/mL (p < 0.05) at 60, 90, and 120 minutes after PLA_2_ injection. In the IPK model, PLA_2_ administration resulted in a nonsignificant increase in IL-5 concentrations in perfusate from 79 ± 4 pg/mL at time 0 to 89 ± 5 and 81 ± 4 pg/mL at the first 10 and 30 minutes, respectively. The concentrations of IL-5 in urine significantly increased throughout the study from 63 ± 7 pg/mL at time 0 to 99 ± 9, 91 ± 7, 95 ± 7, and 96 ± 6 pg/mL (p < 0.05) at 10, 30, 60, and 90 minutes, respectively, after PLA_2_ administration. The percentage of FE_IL-5_ in the intact kidney model decreased to a lower value at 30 minutes (p < 0.05) post-PLA_2_ injection and then gradually increased thereafter near the control level. Conversely, the percentage of FE_IL-5_ significantly increased to a higher value at 30 and 60 minutes (p < 0.05) post-PLA_2_ administration in the IPK model.

The effect of PLA_2_ on the anti-inflammatory cytokine IL-10 is shown in Figure 6C. In the *in vivo* study, concentrations of IL-10 in plasma showed slight increases from 25 ± 1 pg/mL at time 0 to 27 ± 1 and 28 ± 1 pg/mL at 10 and 30 minutes, respectively, whereas IL-10 concentrations in urine increased stepwise from 30 ± 1 pg/mL at time 0 to 39 ± 2 pg/mL (p < 0.05) at 120 minutes after PLA_2_ injection. In the IPK model, PLA_2_ administration showed no alteration in IL-10 concentrations in perfusate, while the concentrations of IL-10 in urine significantly increased from 32 ± 1 pg/mL at time 0 to 39 ± 1 pg/mL (p < 0.05) throughout the study after PLA_2_ administration. The percentage of FE_IL-10_ in the intact kidney model decreased to a lower value at 30 minutes post-PLA_2_ injection and then gradually increased thereafter near the control level. Conversely, the percentage of FE_IL-10_ significantly increased to a higher value at 30 and 60 minutes (p < 0.05) post-PLA_2_ administration in the IPK model.

The effect of PLA_2_ on pro-inflammatory cytokines, specifically IFN-γ, is shown in Figure 6D. In the *in vivo* study, PLA_2_ injection resulted in a significant increase in IFN-γ concentrations in plasma from 22 ± 1 pg/mL at time 0 to 28 ± 1 pg/mL (p < 0.05) at 30 minutes, while concentrations of IFN-γ in urine significantly increased from 7 ± 1 pg/mL at time 0 to 11 ± 0.8 pg/mL (p < 0.05) at 120 minutes post-PLA_2_ injection. In the IPK model, IFN-γ concentrations in perfusate significantly increased stepwise from 6.1 ± 0.6 pg/mL at time 0 to 13.1 ± 1.0 pg/mL (p < 0.05) at 90 min. IFN-γ concentrations in urine showed increases stepwise from 7.3 ± 2.0 to 18 ± 2.0 and 19.0 ± 3.0 pg/mL (p < 0.05) at 60 and 90 minutes after PLA_2_ administration. The percentage of FE_IFN-γ_ in the intact kidney model decreased to a lower value at 30 minutes (p < 0.05) post-PLA_2_ injection and then gradually increased thereafter near the control level. Conversely, the percentage of FE_IFN-γ_ significantly increased to a higher value at 30 and 60 minutes (p < 0.05) post-PLA_2_ administration in the IPK model.

The effect of PLA_2_ on pro-inflammatory cytokines for IL-1β is shown in Figure 6E. Injection of PLA_2_ *in vivo* revealed non-significant decreases in concentrations of IL-1β in plasma from 411 ± 40 pg/mL at time 0 and reaching a nadir of 350 ± 18 pg/mL at 120 minutes post-PLA_2_ injection. The concentrations of IL-1β in urine showed no alteration throughout the study post-PLA_2_ injection. In the IPK model, the concentrations of IL-1β showed slight increases in perfusate from 312 ± 10 pg/mL at time 0 to 340 ± 12 pg/mL at 10 minutes, while the concentrations of IL-1β in urine showed no alteration throughout the study after PLA_2_ administration. The percentage of FE_IL-1β_ in the intact kidney decreased to a lower value at 30 minutes (p < 0.05) post-PLA_2_ injection and then gradually increased thereafter near the control level. Conversely, in the IPK model, the percentage of FE_IL-1β_ increased to a higher value at the first 30 minutes and then gradually decreased thereafter to the end of the experiment after PLA_2_ administration.

The effect of PLA_2_ on pro-inflammatory cytokines, particularly TNF-α, is shown in Figure 6F. In the *in vivo* study, the concentrations of TNF-α in plasma exhibited non-significant decreases throughout the experimental period, starting from 246 ± 30 pg/mL at time 0 and reaching a nadir of 200 ± 25 pg/mL at 120 minutes post-PLA_2_ injection. Similarly, the concentrations of TNF-α in urine also displayed non-significant decreases over the experimental period, declining from 296 ± 45 pg/mL at time 0 to a low point of 212 ± 25 pg/mL at 120 minutes post-PLA_2_ injection. The percentage of FE_TNF-α_ decreased significantly to lower values at 30 minutes (p < 0.05) post-PLA_2_ injection and then returned to control levels in the intact kidney. Conversely, in the IPK model, FE_TNF-α_ significantly increased to higher values at 30 minutes (p < 0.05) post-PLA_2_ injection, followed by a subsequent decrease to the control level.

*The effect of MP on the production of pro-inflammatory and anti-inflammatory cytokines*

**Figure 7.**
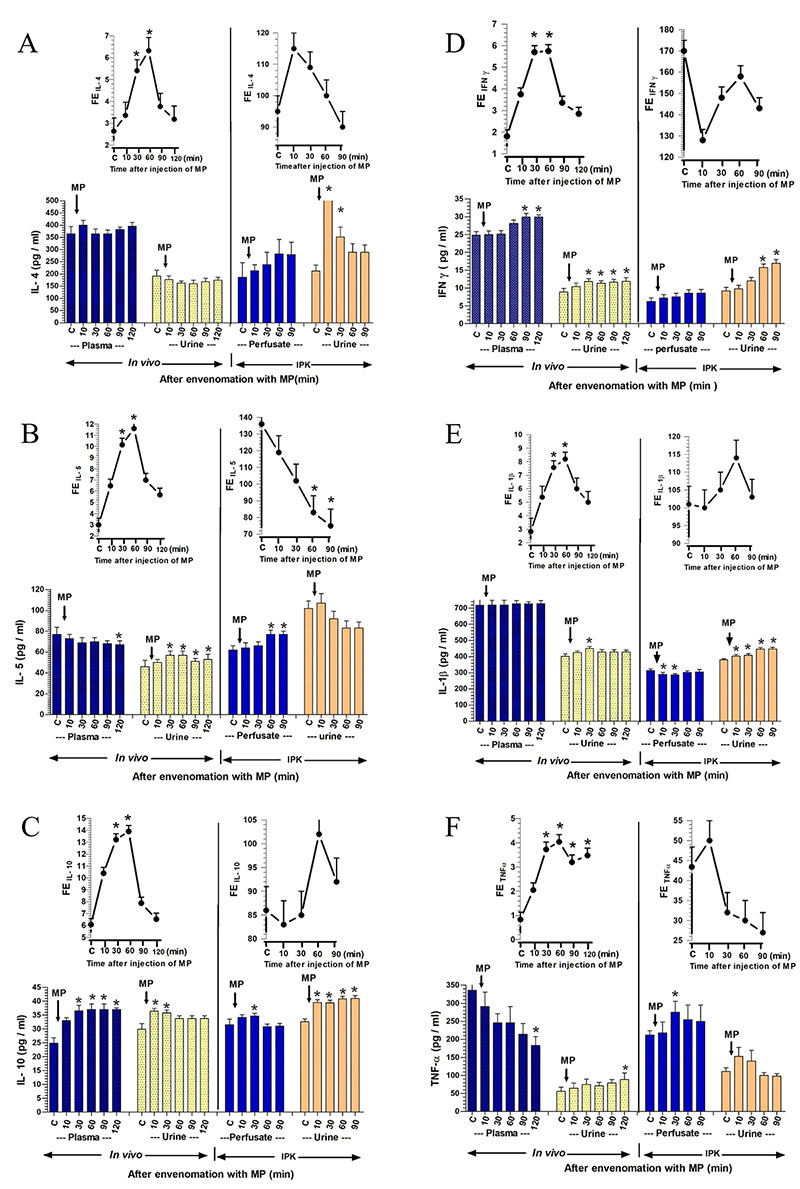
Changes in the concentration of pro-inflammatory and anti-inflammatory cytokines in plasma/perfusate, urine, and urinary fractional excretion (FE) were observed at various time points in response to the administration of MP in *in vivo* and IPK studies. A comparison of two datasets from the treated groups between *in vivo* and the rabbit IPK after the administrations of MP was conducted to analyze changes in **(A)** IL-4, **(B)** IL-5, **(C)** IL-10, **(D)** IFN-γ, **(E)** IL-1β, and **(F)** TNF-α. The data are presented as the mean ± SEM (n = 4). *Significant differences (p < 0.05) were determined using repeated measures ANOVA with Bonferroni post-hoc test, comparing the specified time point to the internal control within the same group.

The effects of MP injection *in vivo* (0.2 mg/kg, i.v.) and in the IPK model (280 µg/100 mL perfusate) on the production of anti-inflammatory and pro-inflammatory cytokines are illustrated in Figure 7. The effect of MP injection on anti-inflammatory cytokines, particularly IL-4 (Figure 7A), demonstrated that IL-4 concentration in plasma showed a non-significant increase from 365 ± 30 pg/mL at time 0 to 400 ± 20 pg/mL at 10 minutes, and maintained non-significant levels throughout the experimental period. Meanwhile, the concentrations of IL-4 in urine exhibited a trend towards a decrease from 191 ± 25 pg/mL at time 0 to a low value of 160 ± 15 pg/mL at 60 minutes after MP injection. In the IPK model, the effect of MP administration showed non-significant increases in IL-4 concentration in perfusate, starting from 180 ± 60 pg/mL at time 0 stepwise to the value of 282 ± 60 pg/mL at 60 minutes after MP administration, while the IL-4 concentration in urine demonstrated significant increases from 212 ± 25 pg/mL at time 0 to 595 ± 40 and 352 ± 40 pg/mL (p < 0.05) at 10 and 30 minutes, respectively after MP administration. The percentage of FE_IL-4_ significantly increased to significant levels (p < 0.05) in the intact kidney at 30 and 60 minutes and gradually decreased thereafter to the control level post-MP injection. Similarly, in the IPK model, FE_IL-4_ increased at 10 minutes after MP administration, gradually decreasing thereafter, approaching the control level.

The effect of MP on anti-inflammatory cytokines, specifically IL-5, is shown in Figure 7B. The concentrations of IL-5 in plasma showed a trend towards a decrease from 79 ± 7 pg/mL at time 0 to a low value of 67 ± 4 pg/mL (p < 0.05) at 120 minutes after MP injection. Conversely, the concentrations of IL-5 in urine demonstrated a significant increase from 46 ± 6 pg/mL at time 0 to 57 ± 4, 57 ± 5, 51 ± 3, and 53 ± 5 pg/mL (p < 0.05) at 30, 60, 90, and 120 minutes, respectively, after MP injection. In the IPK model, MP administration led to significant increases in IL-5 concentration in perfusate, starting from 62 ± 4 pg/mL at time 0 to a value of 77 ± 4 pg/mL (p < 0.05) at both 60 and 90 minutes after MP administration. However, the IL-5 concentration in urine demonstrated a decrease from 102 ± 7 pg/mL at time 0 to 92 ± 7 and 83 ± 6 pg/mL at 30 and both 60 and 90 minutes, respectively, after MP administration. The percentage of FE_IL-5_ in the intact kidney significantly increased to higher values at 30 and 60 minutes (p < 0.05) post-MP injection and then gradually decreased thereafter to the control level. Conversely, the percentage of FE_IL-5_ in the IPK model decreased significantly at 60 and 90 minutes (p < 0.05) post-MP injection.

The effect of MP on the anti-inflammatory cytokine IL-10 is shown in Figure 7C. In the *in vivo* study, concentrations of IL-10 in plasma showed significant increases from 24.8 ± 2 pg/mL at time 0 to 36.5 ± 2 and 37.0 ± 1 pg/mL (p < 0.05) at 30 and 60-120 minutes, respectively. Similarly, IL-10 concentrations in urine significantly increased from 29.2 ± 2 pg/mL at time 0 to 36.4 ± 1 and 35.8 ± 1 pg/mL (p < 0.05) at 10 and 30 minutes, respectively, after MP injection. In the IPK model, MP administration led to significantly increased IL-10 concentrations in perfusate from 31.5 ± 2 pg/mL at time 0 to 35.1 ± 1 pg/mL (p < 0.05), while the concentrations of IL-10 in urine significantly increased from 32.0 ± 1 pg/mL at time 0 to 39.5 ± 1 pg/mL (p < 0.05) throughout the study period after MP administration. The percentage of FE_IL-10_ in the intact kidney model significantly increased to higher values at 30 and 60 minutes (p < 0.05) post-MP injection and then gradually decreased thereafter to the control level. In the IPK model, the percentage of FE_IL-10_ increased to a peak at 60 minutes and then decreased thereafter following MP administration.

The effect of MP on pro-inflammatory cytokines, particularly IFN-γ, is presented in Figure 7D. In the *in vivo* study, concentrations of IFN-γ in plasma showed significant increases from 24.1 ± 1 pg/mL at time 0 to a peak of 30.5 ± 1 and 29.9 ± 1 pg/mL (p < 0.05) at 90 and 120 minutes, respectively, while IFN-γ concentrations in urine significantly increased from 8.9 ± 1 pg/mL at time 0 to 11.8 ± 0.8 pg/mL (p < 0.05) at 30 minutes and throughout the study period after MP injection. In the IPK model, MP administration led to slight increases in IFN-γ concentrations in perfusate from 6.5 ± 1 pg/mL at time 0 to 8.6 ± 1 pg/mL at 90 minutes, while the concentrations of IFN-γ in urine significantly increased from 9.7 ± 1 pg/mL at time 0 to 15.7 ± 1 and 17.0 ± 1 pg/mL (p < 0.05) at 60 and 90 minutes, respectively, after MP administration. The percentage of FE_IFN-γ_ in the intact kidney significantly increased to higher values at 30 and 60 minutes (p < 0.05) post-MP injection and then gradually decreased thereafter to the control level. In the IPK model, the percentage of FE_IFN-γ_ decreased at 10 minutes and then increased thereafter, approaching the control level post-MP administration.

The effect of MP on pro-inflammatory cytokines, specifically IL-1β, is shown in Figure 7E. The concentrations of IL-1β in plasma showed no alteration throughout the study period after MP injection. In contrast, the concentrations of IL-1β in urine increased significantly from 402 ± 15 pg/mL at time 0 to a peak of 450 ± 13 pg/mL (p < 0.05) at 30 minutes post-MP injection. In the IPK model, the concentrations of IL-1β in perfusate significantly decreased from 313 ± 10 at time 0 to 290 ± 12 and 287 ± 7 pg/mL (p < 0.05) at 10 and 30 minutes, respectively, after MP administration, while the concentrations of IL-1β in urine showed progressive significant increases from 381 ± 6 pg/mL at time 0 to a higher value throughout the experimental period, reaching 448 ± 10 pg/mL (p < 0.05) at 90 minutes after MP injection. The percentage of FE_IL-1β_ in the intact kidney significantly increased to higher values at 30 and 60 minutes (p < 0.05) post-MP injection and then gradually decreased thereafter to the control level. Similarly, in the IPK model, the percentage of FE_IL-1β_ increased to a higher value at 60 minutes and then decreased thereafter to the control level post-MP injection.

Finally, the effect of MP on pro-inflammatory cytokines, specifically TNF-α, is presented in Figure 7F. The *in vivo* study demonstrated that the concentrations of TNF-α in plasma progressively decreased significantly from 336 ± 50 pg/mL at time 0, reaching a lower value of 183 ± 25 pg/mL (p < 0.05) at 120 minutes after MP injection. Meanwhile, the concentrations of TNF-α in urine progressively increased significantly from 56 ± 12 pg/mL at time 0 to 89 ± 18 pg/mL (p < 0.05) at 120 minutes after MP injection. In the IPK model, the concentrations of TNF-α in perfusate showed a significant increase from 212 ± 12 pg/mL at time 0 to a peak of 275 ± 31 pg/mL (p < 0.05) at 30 minutes, while the concentrations of TNF-α in urine showed non-significant increases from 111 ± 10 at time 0 to 153 ± 25 and 140 ± 30 pg/mL at 10 and 30 minutes, respectively, after MP injection. The percentage of FE_TNF-α_ increased progressively to significantly higher values at 60 minutes (p < 0.05) after MP injection in the intact kidney, while in the IPK model, FE_TNF-α_ increased in the first 10 minutes and then decreased thereafter throughout 90 minutes following MP administration.

*The effect of LAAO on the production of pro-inflammatory and anti-inflammatory cytokines*

**Figure 8.**
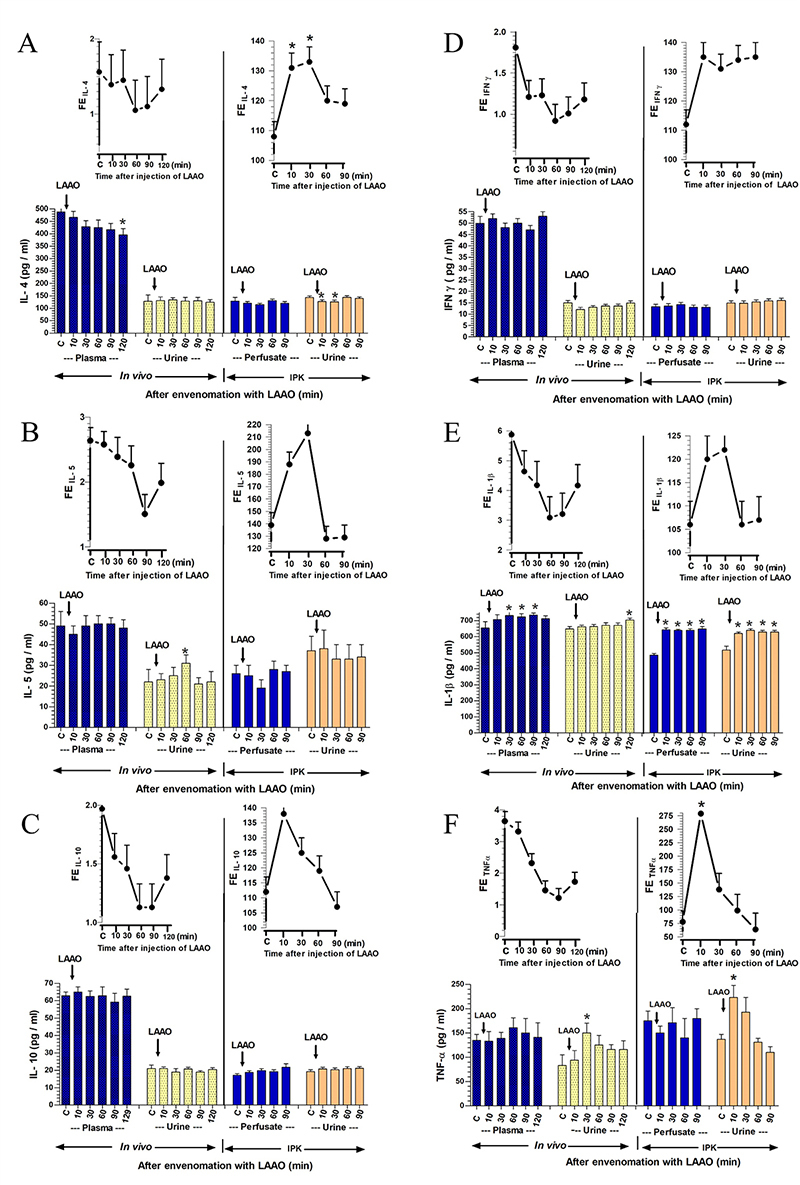
Changes in the concentration of pro-inflammatory and anti-inflammatory cytokines in plasma/perfusate, urine, and urinary fractional excretion (FE) were observed at various time points in response to the administration of LAAO in *in vivo* and IPK studies. A comparison of two datasets from the treated groups between *in vivo* and the rabbit IPK after the administrations of LAAO was conducted to analyze changes in **(A)** IL-4, **(B)** IL-5, **(C)** IL-10, **(D)** IFN-γ, **(E)** IL-1β, and **(F)** TNF-α. The data are presented as the mean ± SEM (n = 4). *Significant differences (p < 0.05) were determined using repeated measures ANOVA with Bonferroni post-hoc test, comparing the specified time point to the internal control within the same group.

The effects of LAAO injection *in vivo* (0.15 mg/kg, i.v.) and in the IPK model (135 µg/100 mL perfusate) on the production of anti-inflammatory and pro-inflammatory cytokines are illustrated in Figure 8. The effect of LAAO on anti-inflammatory cytokines, particularly IL-4, is presented in Figure 8A. In the *in vivo* study, the concentrations of IL-4 in plasma progressively and significantly decreased from 488 ± 25 pg/mL at time 0 to 396 ± 30 pg/mL, reaching a lower value at 120 minutes (p < 0.05), while the concentrations of IL-4 in urine exhibited no significant changes throughout the experimental period after LAAO injection. In the IPK model, the concentrations of IL-4 in perfusate slightly decreased from 129 ± 15 pg/mL at time 0 to 115 ± 15 pg/mL at 30 minutes, while the concentrations of IL-4 in urine showed a significant decrease from 143 ± 7 pg/mL at time 0 to 127 ± 7 and 126 ± 6 pg/mL (p < 0.05) at 10 and 30 minutes, respectively, after LAAO administration. The percentage of FE_IL-4_ slightly decreased post-LAAO injection in the intact kidney, whereas, in the IPK model, FE_IL-4_ increased to a higher value at 10 and 30 minutes (p < 0.05) post-LAAO administration and then gradually decreased to the control level thereafter.

The effect of LAAO on anti-inflammatory cytokines, specifically IL-5, is shown in Figure 8B. In the *in vivo* study, the concentrations of IL-5 in plasma slightly decreased from 49 ± 7 pg/mL at time 0 to 45 ± 4 in the first 10 minutes after LAAO injection. The concentrations of IL-5 in urine significantly increased in the first 60 minutes, rising from 22 ± 6 pg/mL at time 0 to 31 ± 4 pg/mL (p < 0.05) at 60 minutes and then decreased to the control level thereafter after LAAO injection. In the IPK model, the concentrations of IL-5 in perfusate slightly decreased from 26 ± 4 pg/mL at time 0 to 19 ± 4 pg/mL at 30 minutes, while the concentrations of IL-5 in urine showed a slight decrease from 37 ± 7 pg/mL at time 0 to 33 ± 6 pg/mL throughout the study after LAAO administration. The percentage of FE_IL-5_ of the intact kidney progressively decreased to a lower value at 90 minutes post-LAAO injection. Conversely, in the IPK model, the percentage of FE_IL-5_ increased to a higher value at 30 minutes post-LAAO injection and then decreased to the control level after that.

The effect of LAAO on anti-inflammatory cytokines, specifically IL-10, is shown in Figure 8C. The concentrations of IL-10 in both plasma and urine in the *in vivo* study demonstrated no significant alterations after LAAO administration, while the concentrations of IL-10 in perfusate and urine in IPK models also showed no alteration after LAAO administration. The percentage of FE_IL-10_ of the intact kidney decreased to a lower value at 90 minutes post-LAAO injection. In contrast, the percentage of FE_IL-10_ in the IPK model increased to a higher value at 10 minutes post-LAAO injection and then decreased afterward to the control level.

The effect of LAAO on pro-inflammatory cytokines, specifically IFN-γ, is illustrated in Figure 8D. In the *in vivo* study, the concentrations of IFN-γ in plasma showed no significant alterations throughout the study, while the concentrations of IFN-γ in urine slightly decreased from 15 ± 1 pg/mL at time 0 to 12 ± 1 in the first 10 minutes after LAAO injection. In the IPK model, the concentrations of IFN-γ in both perfusate and urine showed no alterations after LAAO administration. The percentage of FE_IFN-γ_ in the intact kidney decreased post-LAAO injection throughout the experimental period, while the percentage of FE_IFN-γ_ in the IPK model showed an increase post-LAAO administration throughout the experimental period.

The effect of LAAO on pro-inflammatory cytokines, specifically IL-1β, is presented in Figure 8E. In the *in vivo* study, concentrations of IL-1β in plasma showed significant increases from 654 ± 40 pg/mL at time 0 to 733 ± 39, 724 ± 20, and 735 ± 12 pg/mL (p < 0.05) at 30, 60, and 90 minutes, respectively, whereas IL-1β concentrations in urine significantly increased from 648 ± 15 pg/mL at time 0 to 704 ± 12 pg/mL (p < 0.05) at 120 minutes after LAAO injection. In the IPK model, LAAO administration showed significantly increased IL-1β concentrations in perfusate from 485 ± 10 pg/mL at time 0 to a peak of 643 ± 12 pg/mL (p < 0.05) at 10 minutes and occurred throughout the study, while the concentrations of IL-1β in urine also significantly increased from 516 ± 25 pg/mL at time 0 to 620 ± 10 pg/mL (p < 0.05) and also occurred throughout the study after LAAO administration. The percentage of FE_IL-1β_ of the intact kidney progressively decreased to a lower value at 60 minutes post-LAAO injection. Conversely, in the IPK model, the percentage of FE_IL-1β_ increased to a higher value at 30 minutes post-LAAO injection and then decreased to the control level after that.

The effect of LAAO on pro-inflammatory cytokines, specifically TNF-α, is shown in Figure 8F. In the *in vivo* study, concentrations of TNF-α in plasma exhibited non-significant increases, rising from 135 ± 12 pg/mL at time 0 to peaks of 161 ± 20, 150 ± 30, and 141 ± 20 pg/mL at 60, 90, and 120 minutes, respectively. Simultaneously, TNF-α concentrations in urine significantly increased from 83 ± 22 pg/mL at time 0 to a peak of 150 ± 20 (p < 0.05), 125 ± 20, and 110 ± 18 pg/mL at 30, 60, and 90-120 minutes after LAAO injection. In the IPK model, TNF-α concentrations in perfusate showed non-significant decreases from 175 ± 20 pg/mL at time 0 to 150 ± 14, 170 ± 31, and 140 ± 40 pg/mL at 10, 30, and 60 minutes, respectively, after LAAO administration. The concentrations of TNF-α in urine significantly increased from 137 ± 10 pg/mL at time 0 to 223 ± 25 (p < 0.05) and 193 ± 30 pg/mL at 10 and 30 minutes, respectively, after LAAO administration, gradually decreasing thereafter near the control level. The percentage of FE_TNF-α_ in the intact kidney decreased in a stepwise approach to a lower value at 90 minutes post-LAAO injection. Conversely, in the IPK model, the percentage of FE_TNF-α_ significantly increased in the first 10 minutes (p < 0.05) post-LAAO administration and then decreased in a stepwise manner near the control level. 

*The effect of PDE on the production of pro-inflammatory and anti-inflammatory cytokines*

**Figure 9.**
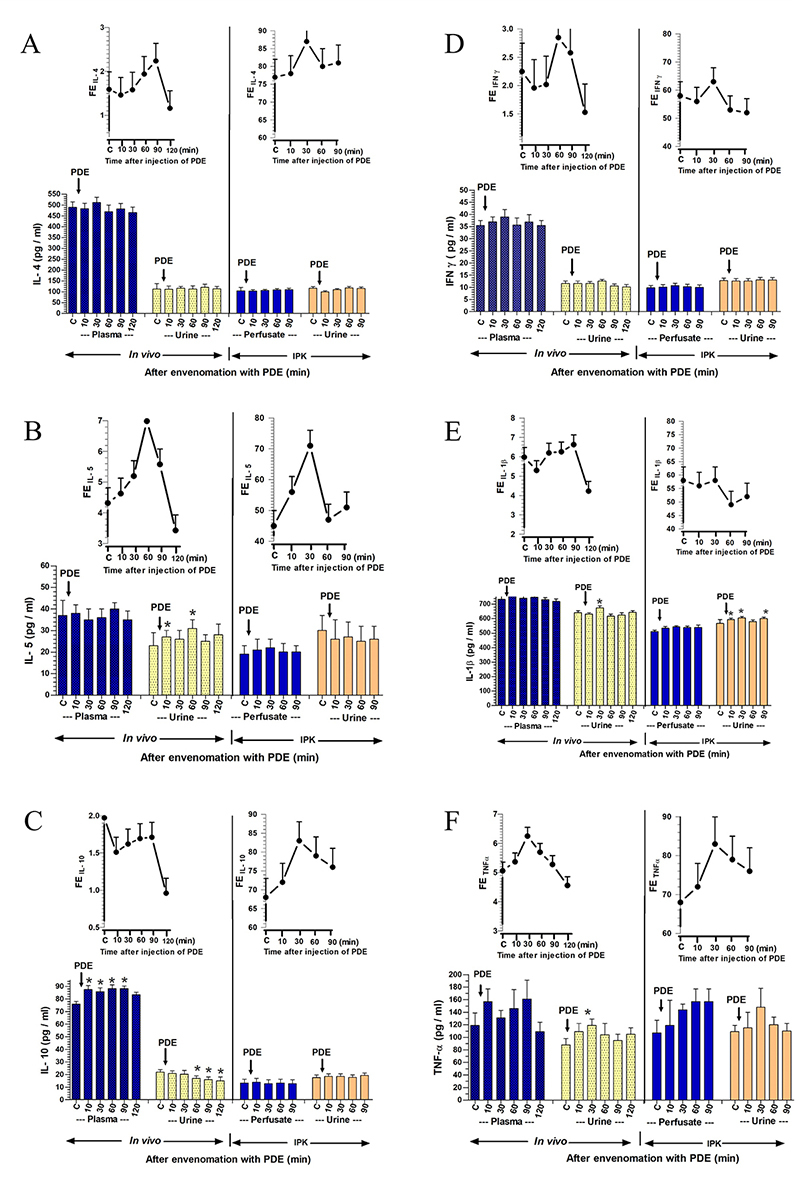
Changes in the concentration of pro-inflammatory and anti-inflammatory cytokines in plasma/perfusate, urine, and urinary fractional excretion (FE) were observed at various time points in response to the administration of PDE in *in vivo* and IPK studies. A comparison of two datasets from the treated groups between *in vivo* and the rabbit IPK after the administrations of PDE was conducted to analyze changes in **(A)** IL-4, **(B)** IL-5, **(C)** IL-10, **(D)** IFN-γ, **(E)** IL-1β, and **(F)** TNF-α. The data are presented as the mean ± SEM (n = 4). *Significant differences (p < 0.05) were determined using repeated measures ANOVA with Bonferroni post-hoc test, comparing the specified time point to the internal control within the same group.

The effects of PDE injection *in vivo* (0.10 mg/kg, i.v.) and in the IPK model (100 µg/100mL perfusate) on the production of anti-inflammatory and pro-inflammatory cytokines are illustrated in Figure 9. The effect of PDE on anti-inflammatory cytokines, particularly IL-4, is shown in Figure 9A. The injection of PDE *in vivo* showed no alterations in the concentrations of IL-4 in either plasma or urine throughout the experimental periods. Similarly, no significant alterations in the concentrations of IL-4 in either perfusate or urine were apparent after the administration of PDE in the IPK model. The percentage of FE_IL-4_ in both the *in vivo* and IPK studies showed no significant alterations in a similar manner after PDE administration. 

The effect of PDE on the anti-inflammatory cytokine IL-5 is shown in Figure 9B. In the *in vivo* study, no significant alterations in the concentrations of IL-5 in plasma were observed after PDE injection throughout the experimental periods, whereas the concentrations of IL-5 in urine from the intact kidney significantly increased from 23 ± 6 pg/mL at time 0 to 27 ± 3 and 31 ± 4 pg/mL (p < 0.05) at 10 and 60 minutes, respectively, after PDE injection. In the IPK model, there were no changes in the concentrations of IL-5 in perfusate, while the concentrations of IL-5 in urine showed non-significant decreases from 30 ± 7 pg/mL at time 0 to 26 ± 6 pg/mL in the first 10 minutes and throughout the subsequent period after PDE administration. The percentage of FE_IL-5_ in the intact kidney increased to a higher value at 60 minutes post-PDE injection and then decreased to the control level thereafter. In the IPK model, the percentage of FE_IL-5_ increased to a higher value at 30 minutes and then decreased to the control level thereafter following PDE administration.

The effect of PDE on the anti-inflammatory cytokine IL-10 is depicted in Figure 9C. Injection of PDE *in vivo* resulted in significant increases in the concentrations of IL-10 in plasma, rising from 76 ± 2 pg/mL at time 0 to a peak of 87 ± 3, 85 ± 3, 88 ± 3, and 88 ± 2 pg/mL (p < 0.05) at 10, 30, 60, and 90 minutes, respectively. Conversely, the concentrations of IL-10 in urine significantly decreased from 22 ± 2 pg/mL at time 0 to 17 ± 2, 16 ± 2, and 15 ± 3 pg/mL (p < 0.05) at 60, 90, and 120 minutes, respectively, after PDE injection. In the IPK studies, no alterations in the concentrations of IL-10 in either perfusate or urine were observed after PDE administration. The percentage of FE_IL-10_ in the intact kidney model decreased to a lower value at 120 minutes post-PDE injection. Conversely, the percentage of FE_IL-10_ in the IPK model increased to a higher value at 30 minutes post-PDE injection.

The effect of PDE on pro-inflammatory cytokines, specifically IFN-γ, is illustrated in Figure 9D. The injection of PDE *in vivo* did not affect the concentrations of IFN-γ in plasma and urine throughout the experimental periods. Similarly, in the IPK model, no significant alterations in the concentrations of IFN-γ in perfusate and urine were observed after administration of PDE. The percentage of FE_IFN-γ_ in both the intact kidney and the IPK model showed a tendency to decrease following PDE injection throughout the experimental period.

The effect of PDE on pro-inflammatory cytokines, particularly IL-1β, as displayed in Figure 9E, showed that injection of PDE *in vivo* resulted in no significant alterations in the concentrations of IL-1β in plasma. In contrast, the concentrations of IL-1β in urine increased from 642 ± 15 pg/mL at time 0 to 675 ± 12 pg/mL (p < 0.05) at 30 minutes after PDE injection. In IPK studies, administration of PDE caused non-significant increases in the concentrations of IL-1β in perfusate, from 501 ± 10 pg/mL at time 0 to 535 ± 12 pg/mL at 10 minutes and throughout all periods of the study, while the concentrations of IL-1β in urine significantly increased from 568 ± 25 pg/mL at time 0 to 594 ± 10, 606 ± 10, and 610 ± 10 pg/mL (p < 0.05) at 10, 30, and 90 minutes, respectively. The percentage of FE_IL-1β_ in both the intact kidney and the IPK model showed no significant decreases post-PDE administration.

The effect of PDE on pro-inflammatory cytokines, particularly TNF-α, is presented in Figure 9F. Injection of PDE *in vivo* resulted in non-significant increases in concentrations of TNF-α in plasma, from 119 ± 20 pg/mL at time 0 to a peak of 157 ± 19, 146 ± 28, and 161 ± 30 pg/mL at 10, 60, and 90 minutes, respectively. The concentrations of TNF-α in urine showed significant increases, rising from 88 ± 10 pg/mL at time 0 to a peak of 119 ± 10 pg/mL (p < 0.05) at 30 minutes after PDE injection. In the IPK model, administration of PDE resulted in increased concentrations of TNF-α in perfusate, from 107 ± 20 pg/mL at time 0 to a peak of 157 ± 20 pg/mL at 60 and 90 minutes after PDE administration, while the concentrations of TNF-α in urine showed a non-significant increase from 109 ± 10 pg/mL at time 0 to a peak of 148 ± 20 pg/mL at 30 minutes after PDE administration. The percentage of FE_TNF-α_ in both the intact kidney and the IPK model exhibited a similar pattern, increasing to a higher value at 30 minutes and then decreasing thereafter to return to control values after PDE injection.

### Effect of RVV or its venom fractions on alterations in the balance of pro-/anti-inflammatory cytokines

*Changes in the ratios of pro-/anti-inflammatory cytokine in urine for TNF-α/IL-4 and TNF-α/IL-10 after RVV or venom fractions administration*

**Figure 10.**
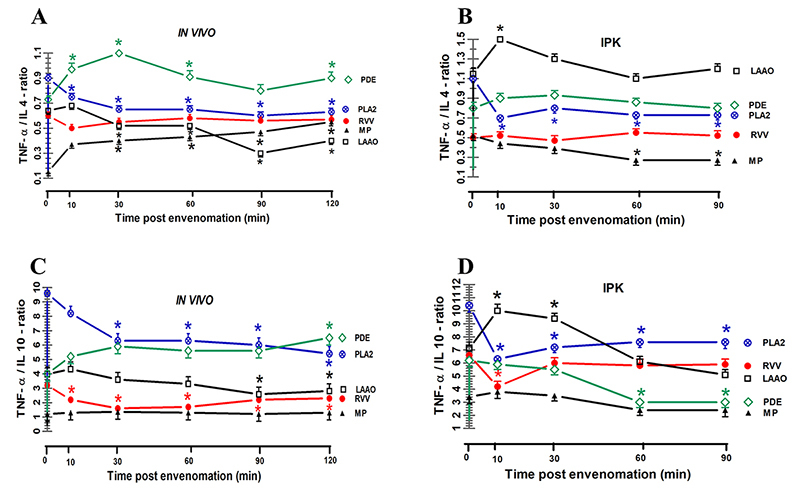
Changes in the pro-inflammatory/anti-inflammatory cytokine balance in urine at various time points in response to the administration of RVV or its venom fractions (PLA_2_, MP, LAAO, and PDE) in both *in vivo* and IPK studies. The ratios of pro-/anti-inflammatory; **(A)** TNF-α/IL-4 for *in vivo* and for **(B)** IPK; **(C)** TNF-α/IL-10 for *in vivo* and for **(D)** IPK. Data are shown as the mean ± SEM (n = 4). *Significant difference (p < 0. 05); repeated measures ANOVA with Bonferroni post-hoc test between the specified time points and the internal control in the same group.

 Cytokine levels in the urine of both the intact kidney (*in vivo*) and the IPK model after the injection of RVV or venom fractions were calculated to investigate the ratios of TNF-α/IL-4 and TNF-α/IL-10, as shown in Figure 10.

As shown in Figure 10A, the TNF-α/IL-4 ratios in the urine of the intact kidney significantly increased (p < 0.05) after the injection of either PDE or MP, whereas the injection of either PLA_2_ or LAAO fraction significantly decreased (p < 0.05), but no alterations were apparent after the injection of crude RVV.

In Figure 10B, the TNF-α/IL-4 ratios in the urine of the IPK gradually increased after the administration of either PDE or RVV, whereas it significantly decreased (p < 0.05) after PLA_2_ or MP administration throughout the experimental periods.

As shown in Figure 10C, the TNF-α/IL-10 ratios in the urine of the intact kidney significantly decreased (p < 0.05) after the injection of RVV, including venom fractions of PLA_2_ or LAAO, whereas it increased gradually after the injection of PDE, but no alterations were apparent after MP injection.

In Figure 10D, the TNF-α/IL-10 ratios in the urine of the IPK model significantly decreased (p < 0.05) after PLA_2_ and PDE administration throughout the experimental periods. The TNF-α/IL-10 ratios in the urine started to significantly decrease at 10 minutes after RVV administration and returned to control levels thereafter. The TNF-α/IL-10 ratios in the urine of the IPK decreased gradually after MP administration throughout the experimental periods, while significant increases in ratios were observed at 10 and 30 minutes (p < 0.05) after LAAO injection.

*Changes in the ratios of pro-/anti-inflammatory cytokine in urine for IFN-γ/IL-4, and IFN-γ/IL-10 after RVV or venom fractions administration*

**Figure 11.**
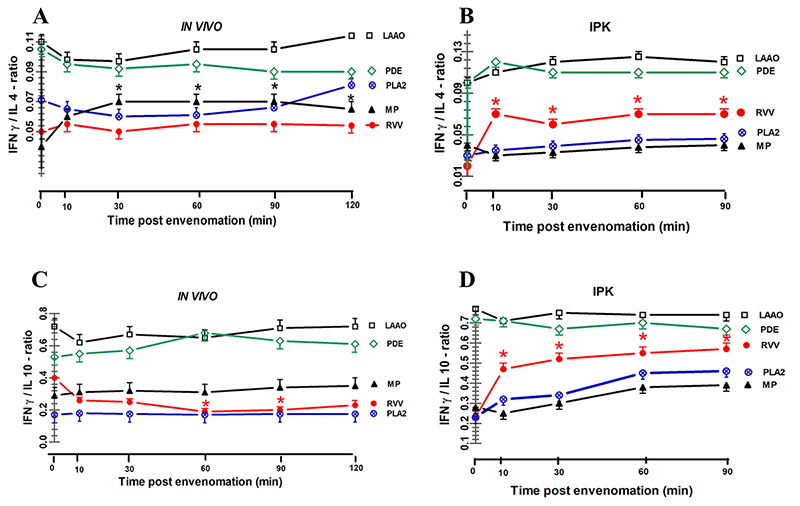
Changes in the pro-inflammatory/anti-inflammatory cytokine balance in urine at various time points in response to the administration of RVV or its venom fractions (PLA_2_, MP, LAAO, and PDE) in both *in vivo* and IPK studies. The ratios of pro-/anti-inflammatory; **(A)** IFN-γ/IL-4 for *in vivo* and for **(B)** IPK; **(C)** IFN-γ/IL-10 for *in vivo* and for **(D)** IPK. Data are shown as the mean ± SEM (n = 4). *Significant difference (p < 0. 05); repeated measures ANOVA with Bonferroni post-hoc test between the specified time points and the internal control in the same group.

 Cytokine levels in the urine of both the intact kidney (*in vivo*) and the IPK model after the injection of RVV or venom fractions were calculated to investigate the ratios of IFN-γ/IL-4 and IFN-γ/IL-10, as shown in Figure 11.

As shown in Figure 11A, the IFN-γ/IL-4 ratios in the urine of the intact kidney significantly increased (p < 0.05) after the injection of MP fractions. However, no alterations in IFN-γ/IL-4 ratios were apparent after the injection of either RVV or venom fractions of PLA_2_, LAAO, and PDE.

In Figure 11B, the IFN-γ/IL-4 ratios in the urine of the IPK significantly increased (p < 0.05) after the administration of RVV throughout the experimental period, whereas no alterations in IFN-γ/IL-4 ratios were observed after the administration of other venom fractions.

As shown in Figure 11C, the IFN-γ/IL-10 ratios in the urine of intact kidneys significantly decreased at 60 and 90 minutes (p < 0.05) after the injection of RVV, while no alterations were observed after the injection of venom fractions.

In Figure 11D, the IFN-γ/IL-10 ratios in the urine of the IPK significantly increased (p < 0.05) after the administration of RVV, while they gradually increased after the administration of venom fractions of PLA_2_ and MP throughout the experimental period. No alterations in IFN-γ/IL-10 ratios were observed after the administration of venom fractions of LAAO and PDE.

## Discussion

The pathophysiological mechanism of AKI induced by *D. siamensis* venom is multifactorial. Two possible factors that may contribute to the risk of development of AKI after envenomation are the late antivenom and insufficient antivenom administration. [10, 11]. Other factors associated with secondary complications; especially oxidative stress caused by venom toxins may persist even after antivenom administration [9]. However, limited data are available regarding the involvement of oxidative stress and inflammatory pathways during envenomation with *D. siamensis* venom. 

### *In vivo* and IPK assessment of pro-oxidant/antioxidant levels in the kidney with the effects of RVV and its venom fractions

The present study was designed to evaluate the effect of RVV on the involvement of oxidative stress and inflammatory pathways in AKI in experimental rabbits. The administration of crude RVV venom and its venom fractions was conducted to investigate early responses in renal functional studies using *in vivo* and IPK models, with a focus on examining the production of mediators associated with oxidative stress and inflammatory cytokines. The results obtained in the present study indicate that the administration of RVV and its venom fractions *in vivo* could initiate systemic and local inflammation, leading to ROS production, as evidenced by the increased lipid peroxidation measured in terms of malondialdehyde (MDA) levels, in plasma, urine, and kidney tissue. The susceptibility of the kidneys to oxidative stress can be attributed to their high oxygen turnover and the presence of long-chain polyunsaturated fatty acids in their lipid composition [8]. The process of lipid peroxidation, with MDA as an end product, serves as a reliable biomarker of oxidative stress that can lead to damage to cellular membranes and injury [33, 34, 35]. Oxidative stress can occur due to an imbalance between antioxidants and prooxidants in the body leading to an increase in ROS and oxidative damage to cells and tissues which can result in irreversible harm to the organ [6, 36]. The administration of RVV or its fractions was found to increase the levels of MDA and antioxidant enzymes, such as SOD, CAT, and GSH in the kidney tissues of both intact and isolated kidneys, indicating a direct effect on kidney tissue with a highly upregulated production of ROS. Venom-induced oxidative stress can arise from an inflammatory response in the affected tissue following a snakebite. This response can involve various mechanisms, including neutrophil chemotaxis to the injury site as a defense mechanism [35], as well as other processes that generate ROS and may trigger the apoptosis process [37]. The IPK study is a useful model for studying the direct effects of toxins on kidney tissue, as it eliminates the confounding factors of systemic factors and endogenous inflammatory mediators. The results of the present study suggest that the RVV and its venom fractions would induce the formation of localized high concentrations of ROS, leading to MDA production, and causing oxidative damage in the kidney tissue. The upregulation of antioxidant enzymes such as SOD, CAT, and GSH may help to neutralize ROS and prevent cell damage. However, the oxidant/antioxidant signaling pathways associated with the effects of treatments involving RVV as well as its venom fractions have not been fully elucidated. The observed variations in antioxidant enzyme levels in the homogenized kidney samples after envenomation suggest different protection mechanisms of endogenous antioxidant enzymes in renal cells against lipid peroxidation in situ. The results of the present study indicate distinct responses in the levels of lipid peroxidation in the homogenized kidney tissue samples during both *in vivo* and IPK studies following the administration of crude RVV and its venom fractions.

It appears that the different venom fractions exert differing effects on lipid peroxidation levels and antioxidant enzyme activity in kidney tissue, highlighting the importance of understanding the specific mechanisms of action of each venom fraction and their potential effects on different organs and tissues. Specifically, the administration of either the RVV or LAAO fraction appeared to have a more pronounced impact on inducing oxidative stress and increasing MDA levels compared to the PLA_2_, MP, and PDE fractions. This may be explained by the fact that LAAO acts exclusively via a separate mechanism of action as a homodimeric flavoenzyme catalyzing the redox reaction of different amino acid groups, thereby generating the catabolic production of H_2_O_2_, keto acids, and ammonia [38]. The H_2_O_2_ generated is highly toxic, leading to the production of reactive oxygen species (ROS) during the enzymatic reaction, which can act on intracellular components and cell membranes. The cytotoxicity caused by LAAO through H_2_O_2_ production triggers autophagy, apoptosis, and necrosis in target cells [39]. These ROS may directly act on cell membranes, leading to altered permeability in the attacked area and inducing endothelial injury [40]. Therefore, it can be argued that the pronounced impact of LAAO in inducing oxidative stress and increasing MDA levels is related to the generation of catabolic production of H_2_O_2_ in the kidney. This suggests that each venom component operates through a distinct mechanism in inducing oxidative stress and inflammation in kidney tissue. These findings align with previous studies indicating that viper venom components can lead to renal toxicity by generating ROS and triggering redox imbalance and oxidative stress [8]. However, the induction of enzymatic antioxidant defenses after exposure to either RVV or venom fractions can be seen as a compensatory mechanism that allows cells to counteract the damage caused by ROS and oxidative stress. This response is important in maintaining the balance between oxidative damage and cellular protection.

The marked elevation of SOD, CAT, and GSH levels in renal tissues after envenomation is likely due to the activation of the antioxidant defense mechanism. The increase in antioxidant enzyme activity of GSH levels may represent an attempt by the body to counteract the damaging effects of venom-induced oxidative stress. It can be hypothesized that the increase in SOD activity presents the first line of defense by directly scavenging the influx of ROS generated in the renal epithelial cells during venom-induced AKI [9]. SOD functions by dismutating the superoxide anion into H_2_O_2_ and O_2_ [41], whereas catalase and glutathione peroxidase convert it into oxygen and water. However, there is a relatively higher degree of both MDA levels and antioxidant enzyme activities of SOD, CAT, and GSH in the intact kidney *in vivo* than in IPK models treated with either RVV or LAAO fraction, compared to those treated with PLA_2_, MP, or PDE. This indicates that different mechanisms of action from different single venom fractions injection cause varying responses in either the local or systemic effect of MDA. Although crude RVV is known to contain a complex mixture of potentially toxic proteins, peptides, and several enzymes [42]. The question then arises as to whether the different changes in oxidation between intact kidney and IPK simply reflect ROS from different sources, playing different roles in the oxidative response, which necessitates precise targeting of ROS that negatively affect the body. Further investigation should be conducted into the physiological processes mediated by ROS scavenging biomaterials to precisely target ROS, which cause a certain degree of damage to the target cell from each venom fraction, either *in vivo* or IPK. The present study shows in various ways that enhancing antioxidants such as SOD, CAT, and GSH levels in the kidney would play a role in combating nephrotoxic agents by blocking lipid peroxidation after envenomation. The decrease in urinary fractional excretion of GSH in the present results indicates a process of compensation aimed at maintaining intracellular antioxidant concentrations and restoring cellular defense mechanisms to protect renal cells after envenomation. An increase in CAT activity has been observed in kidney tissue samples from both *in vivo* and IPK studies after the administration of RVV and its venom fractions. Catalase is an enzyme that is mainly found in peroxisomes and plays a critical role in protecting cells from oxidative damage by dismutating H_2_O_2_ into oxygen and water. This observation supports the findings of an increase in catalase activity in the blood of snakebite patients from the *B. jararacussu* and *B. jararaca* snakes [43]. The increase in CAT activity is linked to the increase in the production of H_2_O_2_, which is a specific substrate for this enzyme [44]. GSH is an endogenous antioxidant that protects against the formation of H_2_O_2_ after envenomation. In kidney tissue samples from *in vivo* and IPK studies treated with RVV and its venom fractions, increased levels of GSH may represent a defensive response to the excessive free radical formation and cellular lysis associated with acute renal injury progression. 

It should be noted that the mechanism of action of venom LAAO fraction involves catalyzing the redox reaction of an L-amino acid to give rise to the production of H_2_O_2_, which can cause oxidative stress and cytotoxicity in target cells [38, 39, 45, 46, 47], indicating that the mode of delivery of this ROS is an important factor in causing high oxidative stress. The separate mechanism of action of LAAO suggests that it may have unique effects on cellular membranes and induce endothelial injury compared to other venom fractions [40].

Other venom components, especially PLA_2_ fraction, belong to a family of ubiquitous enzymes that degrade membrane phospholipids and produce lipid mediators to regulate cellular functions. The action of PLA_2_ can cause cellular injury by disturbing the cell membrane permeability, leading to membrane destabilization through charge and van der Waals interaction [48]. Snake venom PLA_2_ variants have been shown to induce lipid peroxidation by increasing the levels of ROS in venom-induced pathophysiology [9]. Activation of snake venom fraction PLA_2_ results in the hydrolysis of membrane phospholipids and the release of free fatty acids, including arachidonic acid, which serves as a metabolic precursor for important cell signaling eicosanoids [49]. The oxidative metabolism of arachidonic acid also generates ROS. These processes contribute to the formation of lipid peroxides, However, the single injection of venom PLA_2_ fraction showed different results in lower lipid peroxidation response for MDA production compared to the single treatment of crude RVV. These differences may depend on the experimental concentrations of PLA_2_ fraction used since it has been reported that the treatment of serial diluted venom PLA_2_ can remove their overt toxicity, exerting both beneficial and deleterious effects on cell injury [50]. 

The MP venom fraction belongs to a family of zinc-dependent proteases, which are primarily known for their ability to degrade extracellular matrix (ECM) proteins throughout the body, including those in the kidney. On the other hand, the venom MP fraction can cause cellular injury by damaging the cytoskeleton, resulting in the loss of structural integrity of the basement membrane and degradation of extracellular matrix proteins, thereby affecting kidney cell function [51], particularly proteolysis of the ECM and disrupt cell-matrix and cellular adhesion [52]. In addition to its effects on the ECM, MP is also found to co-localize with and proteolyze specific protein targets within renal cells, leading to acute kidney dysfunction. These findings challenge the traditional understanding of MP as an enzyme solely acting on the ECM regarding its activation through non-proteolytic pathways in the presence of increased oxidative stress in the kidney [53].

The effect of a single injection of venom PDE fraction increased MDA concentration in both intact kidneys *in vivo* and IPK studies. The action of venom PDE fraction can cause a reduction in renal hemodynamics [19], which may be due to its hydrolytic activity that breaks down intracellular signaling molecules like cAMP and cGMP [54]. This can lead to a decrease in protection from hypoxemia-induced endothelial injury and oxidative stress, which is known to contribute to a decrease in renal functionality. Several studies have demonstrated that PDE is involved in the mechanisms of AKI [55, 56, 57, 58]. It has been reported that the use of PDE-5 inhibitors (Sildenafil) can protect against*Bothrops alternatus*snake venom (*Ba*V)-induced nephrotoxicity by reduced levels of oxidative stress markers like MDA and GSH [59], suggesting that they may play a protective role against ROS and help maintain oxidant-antioxidant balance [60]. Furthermore, increasing cGMP levels in the kidney has been shown to reduce inflammation and support antioxidant and anti-apoptotic processes [61, 62]. 

### *In vivo* and IPK assessment of pro-oxidant/antioxidant status in relation to renal function and urinary fractional excretion with the effects of RVV and its venom fractions

To more fully establish the link conditions for renal inflammation-venom interactions, we monitored the effects of a single injection of crude venom of RVV or its venom fractions on the renal function, associated excretion of oxidative stress and cytokine levels in the urine in both *in vivo* and IPK studies. This study shows that after crude RVV injection was observed a marked decrease in inulin clearance (GFR) and urine flow in both *in vivo* and the IPK model for two hours after envenomation, which was associated with a marked decrease in systemic blood pressure and a marked increase in RVR. Thus, the reduction of GFR and urine flow would be a consequence of decreased renal perfusion pressure and local vasoconstriction in the kidney. The main part of these pathophysiological mechanisms is orchestrated by the increase of intrarenal vasoconstrictor hormones during envenomation, especially activation of the renin-angiotensin system in response to envenomation with RVV has been reported [63, 64]. In addition, the direct effect of crude RVV administration on the progressive reduction in GFR and urine flow may be mediated by the liberation of the local platelet-activating factor (PAF) [19, 65], the production of thromboxane B_2_ (TxB_2_) [66], which regulates glomerular function by contracting mesangial cell, resulting in a sustained reduction of the glomerular filtration surface and ultrafiltration coefficient (Kf) [67]. 

The effects of the administration of PLA_2_, MP, LAAO, and PDE-containing fractions, on decreases in GFR, urine flow, blood pressure, and an increase in RVR were also observed *in vivo* studies. However, the results demonstrated that the venom fraction results in varying extents as compared to the effect of the crude RVV. These results confirm our previous study [19], indicating that the alterations in renal functions induced by crude RVV are attributed to the synergistic action of various components of snake venom rather than the action of a single component. However, there is limited in vivo data on the specific actions of each venom fraction on how to induce the release of vasoconstrictor hormones and affect renal vasoconstriction after envenomation, which warrants further investigation.

In contrast to the results observed in the IPK model, the administration of each venom fraction resulted in varying extents of changes in the GFR, urine flow, PP, and RVR, throughout the 2-hour study period. The administration of any venom fraction of RVV in the IPK model did not decrease GFR and urine flow including PP and RVR as compared to the effect of injection of crude RVV. These results suggest that alterations in renal hemodynamics caused by RVV and its venom fractions are unlikely to be the primary cause of venom-induced AKI. Instead, the direct action of each venom fraction on renal cells, independent of systemic factors but correlated with their high concentrations, is more likely to be the primary mechanism leading to AKI during treatment. Additionally, the effect of hydrolytic enzymes, particularly PLA_2_ and MP, which are present in high concentrations in viperid snake venom, warrants consideration [42] which may explain these variable effects, as these enzymes act on the cell membrane localization of renal glomeruli, leading to subsequent damage and disruption of the glomerular basement membrane [68]. This disruption increases the permeability of the glomerular filtering membrane, which could increase calculated inulin clearance in this IPK model, leading to progressive increases in GFR and urine flow over time. In addition, in the IPK model, there are neither sympathetic innervations to the IPK nor renin substrate in the system, an interaction of hormonal action on renal vasoconstriction within these systems would not be likely. It indicates that the different venom fractions exert differing effects on IPK, highlighting the importance of understanding the specific mechanisms of the direct action of venom fractions and their potential effects on kidney tissues. Furthermore, it has been evidenced that many pathophysiological phenomena like ischemic conditions secondary to renal vasoconstriction can induce AKI [69, 70, 71], which might not be suspected to occur in the IPK model. 

The direct nephrotoxicity of venom could be partly mediated by lipid peroxidation, which has been suggested as an indicator of AKI [8]. After injections of crude RVV and its venom fraction in both *in vivo* and IPK models, increased MDA and GSH levels were observed in urine and plasma, indicating the direct effects of the venom. End-products of lipid peroxides can leak from the organ or tissue of origin into the bloodstream and can be excreted in urine as well [72, 73, 74]. The detection of these products in urine can be potentially utilized as non-invasive biomarkers of lipid degradation and oxidative stress. The present study provides evidence that detection of MDA level in urine is potentially usable as a late biomarker of oxidative stress and cellular damage in the first 2-3 hours after envenomation with the RVV and its fractions [75].

In this study, the combination of MDA, GSH, and inulin levels in plasma and urine samples were used to calculate the percentage of urinary fractional excretion (FE). The fractional excretion of this biomarker by the kidney can be useful in the evaluation of acute kidney failure following envenomation. These experiments suggest that under oxidative conditions, the renal tubular cell may serve as an impermeable barrier for MDA. We demonstrated that the FE_MDA_ was increased while FE_GSH_ was reduced in both the intact kidney and the IPK model treated with crude RVV for two hours. It indicates that a greater proportion of MDA is excreted in the urine compared to that filtered by the glomeruli at the onset of AKI. In the *in vivo* study, we observed the formation of MDA in urine, likely due to low reabsorption of MDA by the renal tubules. The MDA formation in urine would coincide with the systemic effect of lipid peroxidation while filtered MDA decreases with decreased GFR which may have resulted in an increase in the percentage of FE_MDA_ (C_MDA_/C In x 100) after envenomation. In the IPK model, most of the MDA product following envenomation entered the tubular fluid through tubular secretion. In the IPK model, most of the MDA product following envenomation entered the tubular fluid through tubular secretion, and it was not anticipated for MDA to be reabsorbed by renal tubular cells. During reperfusion, MDA was released from the kidney into the perfusate, leading to an imbalance in the renal tubule between secreted MDA and glomerular-filtered MDA. These experiments suggest that during venom-induced oxidative stress, the renal tubular cell is impermeable to MDA. 

Both intact kidneys and the IPK model exhibited a decrease in FE_GSH_ compared to an increase in GSH levels in both plasma and urine after a single injection of RVV. GSH is a crucial cellular antioxidant that can neutralize the harmful effects of ROS accumulation and remove free peroxides from cells during envenomation [50]. However, in post-envenomation characterized by acute renal and oliguria, the fractional excretion of GSH drops, and, as a consequence, their plasma concentration rises. Therefore, the decrease in FE_GSH_ observed in both intact kidneys and the IPK model would contribute to inflammatory disorders associated with oxidative stress-induced renal injury after RVV administration. A tendency to reduce FE_GSH_ in animals following administrations of either RVV or its venom fractions may lead to decreased renal tubular secretion of GSH, thereby contributing to maintaining intracellular GSH concentrations and restoring cellular defense mechanisms to protect renal cells from lipid peroxidation after envenomation. The different profiles between FE_MDA_ and FE_GSH_ indicate that the tubular handling of GSH and MDA differs. Specifically, the secretion of MDA by renal tubular cells could result from the direct action of venom components on renal tubular epithelial cells. The IPK study demonstrated direct nephrotoxicity without the involvement of systemic factors, as well as lipid peroxidation and subsequent release of MDA from distant organs into plasma and urine. Taken together, these findings suggest that the direct action of venom components on renal tubules would contribute to renal injury, which involves both lipid peroxidation and alterations in GSH metabolism. 

### Assessing pro-inflammatory/anti-inflammatory status *in vivo* and IPK in relation to renal function and urinary fractional excretion with the effects RVV and its venom fractions

The results of the present study indicate that changes in markers of oxidative stress were more pronounced in both the intact kidney and IPK model after administration of RVV and its venom fractions. This suggests that the severity of venom and its components may be linked to the degree of inflammatory activity and impairment in the antioxidant system both initiation and extension of inflammation. The effect of lipid peroxidation has been shown to induce the release of TNF-α from mesangial cells inducing glomerular cell death [76]. TNF-α induces glomerular disease in rabbits [77] and reduces the GFR in the IPK model [78]. Therefore, envenomation-induced AKI would trigger the release of various cytokines, including proinflammatory and anti-inflammatory cytokines, by activating leukocytes and renal tubular cells in the injured kidney [18]. These cytokines serve as sensitive biomarkers and play crucial roles in mediating both the initiation and extension of inflammation. In the present *in vivo* study, the levels of proinflammatory cytokines, namely IL-1β, IFN-γ, and TNF-α, in the plasma and urine were evaluated. Different responses were observed between the actions of the crude RVV and its venom fractions, leading to an inflammatory cascade within 120 minutes after envenomation. These cytokines play a crucial role in triggering a robust defense against external pathogens, aided by anti-inflammatory cytokines such as IL-4, IL-5, and IL-10, which help regulate the inflammatory response. However, the production of pro-inflammatory and anti-inflammatory cytokines would be strictly controlled by complex feedback mechanisms and excessive production of these mediators may significantly contribute to multiple organ failure and death [79, 80]. The present results of the *in vivo* study demonstrated that two types of proinflammatory cytokines, IFN-γ and TNF-α, were increased in plasma, while IL-1β did not show the proinflammatory burden and was decreased in plasma after a single injection of LD_50_ of RVV. An elevation in plasma IFN-γ concentration was observed in all groups of rabbits following a single administration of either crude RVV or its venom fractions. These findings may support evidence that IFN-γ is produced by a variety of cell types and probably plays a role in the early stages of the host response to venoms. Additionally, in the groups of rabbits injected with either MP or PLA_2_, the levels of IFN-γ were significantly increased in both urine and plasma throughout the 120 minutes after administration. The changes in plasma levels of IFN-γ after envenomation in all groups were observed to increase, indicating that IFN-γ is a proinflammatory cytokine that plays a crucial role in host defense. This cytokine also can modulate the inflammatory response by up-regulating various proinflammatory mediators, such as TNF-α and IL-1β [81]. TNF-α has been demonstrated to induce glomerular dysfunction and decrease the glomerular filtration rate (GFR) in isolated perfused rabbit kidneys [77, 78, 79]. The present findings suggest that the functional characteristics of IFN-γ bioactivity play a vital role as a mediator in the inflammatory process of snake envenomation, exhibiting similarities to various models of inflammatory diseases. [81].

Under the conditions used in the IPK study, we observed an increase in IFN-γ concentrations in urine and perfusate following the injection of either RVV or its venom fractions. This indicates that administration of either RVV or its venom fractions directly affected kidney tissues, leading to the induction of IFN-γ production. As AKI is characterized by renal tubule injury following envenomation [19], the elevation in urine IFN-γ concentration in AKI may be attributed to impaired renal proximal tubular reabsorption of glomerular-filtered IFN-γ. This suggests that IFN-γ acts as a pro-inflammatory cytokine and can modulate the inflammatory response. Moreover, this observation is significant in the absence of other inflammatory cytokines.

TNF-α is a cytokine protein that exists in both soluble and transmembrane forms as a primary mediator of the systemic inflammatory response syndrome (SIRS) and is believed to be involved in mediating renal insufficiency in various renal conditions following envenoming [82]. The release of TNF-α in response to RVV and its venom fractions administrations showed different pattern responses. The release of TNF-α in response to RVV and its venom fractions administrations showed similar patterns of responses in the levels of TNF-α in both plasma and urine between *in vivo* and IPK studies. In *in vivo* studies, RVV and its venom fractions have been shown to induce the production of TNF-α, primarily from macrophages capable of synthesizing and releasing TNF-α into circulation [18]. An increase in plasma concentrations of TNF-α was apparent within the first 30 minutes and then declined thereafter after a single injection of either RVV or venom fractions of LAAO and PDE. However, the concentrations of TNF-α in plasma decreased after injection of PLA_2_ or MP fraction. Under the similar conditions used in the present study, we observed that the injection with PLA_2_ or MP fraction would have significant increases in IL-10 levels in both plasma and urine in groups of rabbits. This suggests that the release of TNF-α in plasma, primarily produced by macrophages in response to various stimuli during envenomation, plays a crucial role in initiating a robust defense against external toxins. This process is facilitated by inflammatory mediators through the elevation of the anti-inflammatory cytokine IL-10, which is involved in the regulation of inflammatory responses [83].

The release of TNF-α and IL-1β, into the circulation after envenomation has also been demonstrated in other experimental animals injected with *Bothrops* venom [84, 85] and *Vipera russelli* venom [9]. Barraviera et. al [86] described, for the first time, a systemic inflammatory response syndrome in humans bitten by Brazilian venomous snakes. It is an acute phase reaction with massive release of pro-inflammatory cytokines (particularly IL-1, IL-6, and TNF-α) and acute phase proteins, especially C-reactive protein (CRP). These findings were recently corroborated by Paulino and Di Nicola [87]. In addition, the investigation of renal function indicates that the kidney primarily filters smaller pro-inflammatory cytokines (< 20 kd) at the glomerulus, while larger anti-inflammatory cytokines (> 20 kd) are filtered to a lesser extent. These pro-inflammatory cytokines are then presented to the proximal renal tubules. Generally, the smaller pro-inflammatory cytokines may not be excreted in the urine as they are absorbed by the proximal tubular cells and denatured by intracellular proteolytic mechanisms [88, 89]. The larger anti-inflammatory cytokines (IL-4, IL-5, and IL-10), which typically counterbalance the effects of the smaller pro-inflammatory cytokines, pass less readily into the glomerular filtrate. Lower excretion of urinary concentrations of TNF-α, IFN-γ, and IL-1β was observed after a single injection of RVV, suggesting that the magnitude of the pro-inflammatory response during envenomation may correlate with the magnitude of proximal tubular injury. It is conceivable that low proinflammatory IL-1β levels in plasma would be evident *in vivo* when using RVV and venom fraction of PLA_2_. These findings are consistent with the report that injection with *B. asper* venom in mice did not detect any increments of IL-1β in the serum [90]. In contrast, other studies employing the same *B. asper* venom has reported that an increase in IL-1β in the serum was apparent in mice injected with one LD_50_ venom [85]. This response differed from the IFN-γ response, and the concentration of IL-1β in plasma progressively declined, suggesting that these differences may be attributed to the response of its anti-inflammatory cytokine activities such as IL-4, IL-5, IL-10 via inhibition of the expression of IL-1β, and TNF-α [91] or as the classical pro-inflammatory cytokine cascade observed during acute inflammation, such as sepsis [92, 93].

However, some studies have shown a cytotoxic effect of pro-inflammatory cytokines like TNF-α being proposed through enhanced synthesis of tissue-damaging substances, such as nitric oxide in leukocytes [94, 95]. The pro-inflammatory cytokines IL-1β, TNF-α, and IFN-γ have been shown to induce the inducible nitric oxide synthase (iNOS) in human proximal tubular cell culture, and the resulting nitric oxide is considered nephrotoxic. The time-dependent induction of iNOS is proposed as a mechanism of pro-inflammatory cytokine-induced proximal tubular damage [96]. The reason for this is not clear, since our previous study in the IPK model, which has been recognized as a suitable model for the study of renal functions, did not show any significant changes in urinary NO concentrations at any point in all the RVV and its venom fractions-treated groups during the 90-minute perfusion period [19]. Therefore, it indicates that the mechanism of pro-inflammatory cytokines plays a role that is independent of signal transduction pathways for NO production. A direct role for NO in AKI during the envenomation of crude RVV and its venom fractions in the IPK model can be ruled out [19]. 

To gain a deeper understanding of the inflammatory processes potentially implicated in the local effects induced by RVV and its venom fractions, we analyzed the release of both proinflammatory and anti-inflammatory cytokines in the urine of intact kidneys and in IPK rabbits. The role of the kidney in the clearance of the inflammatory cytokines after envenomation was evaluated. The fractional excretion of cytokine was calculated as a part of the evaluation of acute renal failure. The percentage fractional excretion (FE) of inflammatory cytokines (C_cytokine_/C_In_ x 100, where C_cytokine_ is the filtered cytokine load excreted) has been used as indices of reflecting proximal tubular function in acute renal failure. Our suggestion aligns with studies indicating that filtration of proinflammatory cytokines with small molecular weights would be reabsorbed at the renal proximal tubule [97]. Proximal reabsorption of cytokines increases when renal perfusion and GFR decrease. Fractional excretion of inflammatory cytokines would relate inversely to the proximal tubule reabsorption of cytokines. Thus, renal tubular reabsorption of pro-inflammatory cytokines such as TNF-α and IFN-γ leads to a progressive decrease in FE _TNF-α_ and FE _IFN-γ_ after administration of RVV and its venom fractions for PLA_2_, LAAO, and PDE except for MP. Fractional excretion of inflammatory cytokines showed a progressive decrease after venom fraction MP administration *in vivo*. It is possible that the effect of MP on degradation of the ECM proteins would lead to loss of the basement membrane structural and functional integrity, and so affect the kidney cells, particularly a proximal tubular injury [19]. It would lead to impaired renal tubular epithelium function, reducing its ability to deactivate and reabsorb several inflammatory cytokines, thereby contributing to elevated renal fractional excretion levels. Therefore, in the intact kidney, the decreased clearance and altered renal excretion of these cytokines may contribute to the exaggerated inflammatory response observed in AKI.

However, the shapes of curves representing the urinary fractional excretion of cytokines differ between *in vivo* and *ex vivo* studies following the injection of RVV and its venom fractions. It is known that the IPK operates in a cell-free medium, devoid of interference from systemic factors. This eliminates the involvement of endogenous inflammatory mediators not present in this system. Therefore, the administration of RVV and its venom fractions could directly stimulate the kidney to release both pro-inflammatory and anti-inflammatory cytokines into the urine and perfusate, augmenting the filtered cytokine load within the renal perfusion system without the influence of other signal transduction mediators. The release of inflammatory cytokines from the kidney itself into urine and their accumulation in the perfusate system would cause a progressive increase in the percentage of the filtered cytokine load excreted in the IPK model.

We demonstrated that the direct effect of either RVV or its venom fractions results in a more pronounced release of proinflammatory cytokines, especially IFN-γ and TNF-α. The feedback regulation of the inflammatory response involves the release of anti-inflammatory cytokines (IL-4, IL-5, and IL-10) in the kidney into the urine, as noted after injections of RVV and its venom fractions [18]. 

The significant secretion of both pro-inflammatory and anti-inflammatory cytokines induced by RVV and its venom fractions in the kidneys suggests a direct mechanism that initiates an acute-phase response, likely due to localized tissue damage [90]. It is conceivable that glomerular-filtered proinflammatory cytokines could harm the tubular cells of the kidney, thereby contributing to ongoing renal dysfunction even after antivenom administration in the case of snakebite. In our previous study using the IPK model, we provided evidence that the injection of a 2LD_50_ of crude RVV directly causes nephrotoxic effects, characterized by lesions in the glomerular region and tubulonephrosis [19, 24]. 

IL-4 is a multifunctional cytokine for its anti-inflammatory properties, particularly its role in inducing Ig class switching in response to various stimuli *in vivo* [98]. The present study showed that RVV can stimulate IL-4 production in plasma except for its venom fractions. The IPK model showed that the injection of RVV and its venom fractions for PLA_2_ and MP increase IL-4 concentrations in both urine and perfusate which certainly is exerting a modulatory effect of kidney inflammatory response. IL-4 functions as a natural antagonist cytokine, competing with IL-1β for receptor binding without initiating signal transduction. Despite the possible release of IL-1β in restricted amounts after envenomation, the elevated concentration of IL-4 seems to proactively dampen the bioactivity of IL-1β, at least within the circulation [99]. 

IL-10 is a pluripotent immunoregulatory cytokine that acts as an anti-inflammatory cytokine, potentially inhibiting the secretion of proinflammatory cytokines such as TNF and IL-1β [83], and regulating the differentiation and proliferation of several immune cells [100]. In the present study, either *in vivo* or IPK studies showed that a single injection of RVV and venom fraction of either PLA_2_ or MP can increase the concentration of IL-10 and IL-5, in both plasma and urine at 10-30 minutes. It would suggest that both anti-inflammatory cytokines for IL-5 and IL-10 play a role in various kidney diseases by activating an anti-inflammatory response, immunoregulation, and relieving kidney tissue fibrosis. IL-10 has also been shown to cause immunosuppression associated with various forms of trauma by attenuating TNF-α and IL-1β while enhancing IFN-γ production in plasma and urine [101]. These cytokines are released into the blood or urine and may serve as biomarkers of early AKI. However, the regulatory mechanism among these responses is unclear, as research on the effect of IL-10 on different kidney diseases mainly focuses on in vitro and animal experiments [102].

### *In vivo* and IPK assessment of the proinflammatory and anti-inflammatory balance in the urine with the effects of RVV and its venom fractions

A balanced ratio of pro- and anti-inflammatory cytokines holds significance in assessing the inflammatory status of renal cells, indicating the risk of excessive inflammation or hyporesponsiveness, both of which can result in complications during envenoming. The current study illustrates the equilibrium between concentrations of pro-inflammatory and anti-inflammatory cytokines in urine. This equilibrium potentially reflects the inflammatory status of kidney cells during envenoming processes within two hours post-administration of RVV and its venom fractions. Kidney function following envenomation was evaluated to estimate the inflammatory status, along with exploring the correlation between urinary TNF-α/IL-10 ratio, TNF-α/IL-4 ratio, IFN-γ/IL-10 ratio, and IFN-γ/IL-4 ratio, which could serve as indicators for early AKI. Given the kidney's role in eliminating small, easily filtered proinflammatory cytokines from circulation and neutralizing them with larger anti-inflammatory cytokines, it can effectively maintain intrarenal cytokine balance and promptly trigger a robust endogenous protective anti-inflammatory cytokine response in urine.

The results of the *in vivo* study displayed significant progressive decreases in both TNF-α/IL-10 and IFN-γ/IL-10 ratios following a single RVV injection. Conversely, a single injection of venom fractions containing PLA_2_ and LAAO demonstrated significant progressive decreases in both TNF-α/IL-10 and TNF-α/IL-4 ratios. However, no changes in the balanced ratios of TNF-α/IL-4 and IFN-γ/IL-4 were evident after RVV envenoming, However, no significant changes were observed in the balanced ratios of TNF-α/IL-4 and IFN-γ/IL-4 following RVV envenoming in vivo. Conversely, significant decreases were noted in the balanced ratios of TNF-α/IL-10 and IFN-γ/IL-10, suggesting that IL-10 played a significantly more substantial role as the predominant type of anti-inflammatory cytokine compared to IL-4 during the initiation of intrarenal anti-inflammatory responses following the injection of RVV and its venom fractions. The elevation of urinary IL-10, indicating an anti-inflammatory dominance that aids in the secure elimination of proinflammatory cytokines from filtered plasma into the urine, could signify heightened renal tubular injury after a single injection of crude RVV. The high level of urinary IL-10 causes the deviation of balance, resulting in a notable reduction in either the TNFα/IL-10 ratio or the IFN-γ/IL-10 ratio. However, different responses of inflammatory cytokine by different RVV venom fractions would be due to different mechanisms of action [19] that result in varying levels of pro- and anti-inflammatory cytokine activity, either locally or systemically.

The study conducted on the IPK model demonstrated that the injection of RVV and its venom fractions led to an increase in both urinary pro-inflammatory and anti-inflammatory cytokines. These findings may support evidence showing that RVV and its venom fractions directly influenced the injured renal cells themselves to secrete cytokines [18], such as TNF-α, IFN-γ, IL-1β, IL-4, IL-5, and IL-10, into the urine, including the perfusion system. These cytokines share mechanisms of a responsive state that contribute to changes in urinary cytokine balance and renal tubular dysfunction. The injection of venom fractions containing PLA_2_ and MP in the IPK model elevated both urinary IL-10 and IL-4, leading to a disruption in cytokine balance, resulting in a notable reduction in both the TNF-α/IL-10 ratio and the TNF-α/IL-4 ratio. Conversely, the single injection of RVV caused an increase in urinary IFNγ, shifting the cytokine balance and resulting in significant increases in both the IFNγ/IL-10 ratio and the IFN-γ/IL-4 ratio in the IPK model.

## Conclusion

The experimental studies, both *in vivo* and *ex vivo*, revealed that AKI pathogenesis is related to various and complex mechanisms that lead to the adoption of a combination strategy. Injection of RVV and its venom fractions can initiate systemic and local inflammation associated with oxidative stress and the inflammatory pathway. The parallel presence of two pathways in the kidney after envenomation complicates the pathophysiology of induced AKI. Our data revealed that the lipid peroxidation product, marked increases in the oxidative biomarker, especially MDA levels in both plasma and urine, occur after the injection of either RVV or its venom fractions, both in vivo and in the IPK model. These results suggest that RVV-induced AKI is related to a remarkably enhanced rapid production of lipid peroxidation products, spreading oxidative damage after 2-3 hours of envenomation. The bidirectional effect of the venom affects both oxidative stress and inflammatory cytokines, which can induce AKI through kidney tissue damage. Our results also provide evidence that changes in urinary cytokine concentrations differ from plasma concentrations, especially within the first three hours after envenomation, potentially contributing to the kinetics of certain cytokines. Urinary cytokines could serve as sensitive biomarkers for assessing the impact of RVV and its venom fractions on renal damage and inflammation shortly after envenomation. The effect of RVV or its venom fractions is regulated by both pro- and anti-inflammatory cytokine responses. In groups of rabbits *in vivo* injected with RVV or its venom fractions for a short period, the deviation from the pro-/anti-inflammatory balance in urine shifted towards an anti-inflammatory dominance, as opposed to the pro-inflammatory predominant type within the first two hours post-RVV. In contrast to the IPK model, injection of RVV or its venom fractions resulted in a deviation towards pro-inflammatory dominance. Thus, alterations in renal function following RVV envenomation depend on synergistic actions among various venom components. 

### Abbreviations

AKI: acute kidney injury; ARF: acute renal failure; BP: arterial blood pressure; BHT: butylated hydroxytoluene; CAT: catalase; Cin: Inulin clearance; CRP: C-reactive protein; DTNB: 5,5-dithio-bis-(2-nitrobenzoic acid); ECM: extracellular matrix proteins; FE_GSH_: fractional glutathione reductase excretion; FE_IFN-γ_: fractional interferon gamma excretion; FE_IL-1β_: fractional interleukin-1 beta excretion; FE_IL-4_: fractional interleukin 4 excretion; FE_IL-5_: fractional interleukin 5 excretion; FE_IL-10_: fractional interleukin 10 excretion; FE_MDA_: fractional malondialdehyde excretion; FE_TNF-α_: fractional tumor necrosis factor alpha excretion; GFR: glomerular filtration rate; GSH: reduced glutathione, glutathione reductase; H_2_O_2_: hydrogen peroxide; IFN-γ: Interferon gamma; IL-1β: Interleukin-1 beta; IL-4: Interleukin 4; IL-5: Interleukin 5; IL-10: Interleukin 10; In: Inulin; iNOS: inducible nitric oxide synthase; IV: intravenous injection; IPK: isolated perfused rabbit kidney; Kf: ultrafiltration coefficient;; LAAO: L-amino acid oxidase; MAP: mean arterial blood pressure; MDA: Malondialdehyde; MKHS: modified Krebs-Henseleit Saline solution; MP: metalloproteinase; NO: nitric oxide; PAH: p-amino hippuric acid; PDE: phosphodiesterase; PLA_2_: phospholipase A_2_ ; PP: perfusion pressure; ROS: reactive oxygen species; RVR: renal vascular resistance; RVV: Russell’s viper (*Daboia siamensis*) venom; SOD: superoxide dismutase; TBARS: thiobarbituric acid-reactive substances; TNF-α: Tumor necrosis factor alpha; TxB_2_: Thromboxane B_2_.
